# Oridonin promotes endoplasmic reticulum stress via TP53-repressed TCF4 transactivation in colorectal cancer

**DOI:** 10.1186/s13046-023-02702-4

**Published:** 2023-06-19

**Authors:** Fangyuan Zhou, Haiyang Gao, Luorui Shang, Jinxiao Li, Mengqi Zhang, Shuhan Wang, Runze Li, Lin Ye, Shenglan Yang

**Affiliations:** 1 Department of Integrated Traditional Chinese and Western Medicine, Union Hospital, Tongji Medical College, Huazhong University of Science and Technology, Hubei Province, 1277 Jiefang Avenue, Wuhan, 430022 China; 2 Department of Gastrointestinal Surgery, Union Hospital, Tongji Medical College, Huazhong University of Science and Technology, Hubei Province, 1277 Jiefang Avenue, Wuhan, 430022 China; 3 Clinical Nutrition Department, Union Hospital, Tongji Medical College, Huazhong University of Science and Technology, Hubei Province, 1277 Jiefang Avenue, Wuhan, 430022 China

**Keywords:** Endoplasmic reticulum stress, Oridonin, Transcription factor 4, Tumor protein p53, Colorectal cancer

## Abstract

**Background:**

The incidence of colorectal cancer and cancer death rate are increasing every year, and the affected population is becoming younger. Traditional Chinese medicine therapy has a unique effect in prolonging survival time and improving the prognosis of patients with colorectal cancer. Oridonin has been reported to have anti-cancer effects in a variety of tumors, but the exact mechanism remains to be investigated.

**Methods:**

Cell Counting Kit-8 assay (CCK8) and 5-Ethynyl-2'-deoxyuridine (EdU) staining assay, Tranwell, and Wound healing assays were performed to measure cell proliferation, invasion, and migration capacities, respectively. The protein and mRNA expression levels of various molecules were reflected by Western blot and Reverse Transcription quantitative Polymerase Chain Reaction (qRT-PCR). Transcription Factor 4 (TCF4) and its target genes were analyzed by Position Weight Matrices (PWMs) software and the Gene Expression Omnibus (GEO) database. Immunofluorescence (IF) was performed to visualize the expression and position of Endoplasmic Reticulum (ER) stress biomarkers. The morphology of the ER was demonstrated by the ER tracker-red. Reactive Oxygen Species (ROS) levels were measured using a flow cytometer (FCM) or fluorescent staining. Calcium ion (Ca^2+^) concentration was quantified by Fluo-3 AM staining. Athymic nude mice were modeled with subcutaneous xenografts.

**Results:**

Oridonin inhibited the proliferation, invasion, and migration of colorectal cancer, and this effect was weakened in a concentration-dependent manner by ER stress inhibitors. In addition, oridonin-induced colorectal tumor cells showed increased expression of ER stress biomarkers, loose morphology of ER, increased vesicles, and irregular shape. TCF4 was identified as a regulator of ER stress by PWMs software and GEO survival analysis. In vitro and in vivo experiments confirmed that TCF4 inhibited ER stress, reduced ROS production, and maintained Ca^2+^ homeostasis. In addition, oridonin also activated TP53 and inhibited TCF4 transactivation, further exacerbating the elevated ROS levels and calcium ion release in tumor cells and inhibiting tumorigenesis in colorectal cancer cells in vivo.

**Conclusions:**

Oridonin upregulated TP53, inhibited TCF4 transactivation, and induced ER stress dysregulation in tumor cells, promoting colorectal cancer cell death. Therefore, TCF4 may be one of the important nodes for tumor cells to regulate ER stress and maintain protein synthesis homeostasis. And the inhibition of the TP53/TCF4 axis plays a key role in the anti-cancer effects of oridonin.

**Supplementary Information:**

The online version contains supplementary material available at 10.1186/s13046-023-02702-4.

## Introduction

Colorectal cancer (CRC) is the third most commonly diagnosed cancer worldwide and one of the leading causes of cancer-related mortality [1, 2]. Diet, genetics, polyp lesions, and chronic inflammation all contribute to the high incidence of colorectal cancer [3]. Additionally, it has been demonstrated that the incidence of colorectal cancer is becoming younger [4]. Chinese medicine is emerging widely in colorectal cancer treatment options because of its unique advantages in relieving symptoms, prolonging survival, modulating immune function, and improving life quality [5, 6]. Determining the mechanism of tumor suppression in Chinese medicine has therefore become crucial.

Oridonin, a diterpenoid compound extracted from *Rabdosia rubescens*, has been shown to have anticancer effects on various tumors [7–9]. Oridonin is reported to promote apoptosis and autophagy in tumors by inhibiting nuclear factor-kappa B (NF-kappa B) activation and inducing G2/M phase block [10–13]. Moreover, oridonin targets Caspase-9 to regulate oxidative stress [14, 15] and activates PI3K/Akt and TP53-related pathways to stimulate Reactive Oxygen Species (ROS) production [16], all of which effectively limit tumor growth. Moreover, elevated cytoplasmic ROS levels have also been reported as a key early event in the Endoplasmic Reticulum stress response and apoptosis induced by oridonin in laryngeal squamous cell carcinoma (LSCC) cells [17]. These clues aroused our interest in oridonin and Endoplasmic Reticulum stress, and we wanted to further explore the correlation between oridonin and Endoplasmic Reticulum stress and clarify the regulatory effects between them.

Endoplasmic Reticulum stress is executed to correct misfolded and unfolded protein aggregates in the lumen of the Endoplasmic Reticulum and disturbed Ca^2+^ homeostasis caused by multiple oncogenic stresses. However, the continuous stress response can lead to processes such as Caspase-12-mediated apoptosis, resulting in cell death [18, 19]. Current research on its anticancer mechanisms focuses on endogenous apoptosis triggered by the Endoplasmic Reticulum stress excess [20]. Eukaryotic translation initiation factor 2 alpha kinase 3 (EIF2AK3/PERK), Endoplasmic Reticulum to nucleus signaling 1-alpha (IRE1α), and activating transcription factor 6 (ATF6) are used as Endoplasmic Reticulum stress sensors [21]. When Endoplasmic Reticulum stress occurs, PERK or IRE1α is activated by phosphorylation and affects cell fate via the p-PERK/ATF4/CHOP pathway or p-IRE1α/JNK/Caspase-12 pathway, inducing it to cell autophagy or apoptosis [22–25]. In addition, ATF6 is translocated to the Golgi apparatus and is hydrolyzed to undergo nuclear translocation, inducing cellular autophagy through the upregulation of DNA damage-inducible transcript 3 (DDIT3/CHOP) transcript levels [26]. The induction of ROS production and autophagy by Oridonin has led us to question whether there is a link between Oridonin and Endoplasmic Reticulum stress.

Tumor protein p53 (TP53) is associated with a variety of transcriptional and non-transcriptional activities in the cancer process that exert tight control over cellular senescence, proliferation, DNA damage, repair, and cell death [27]. Inactivating mutations in the TP53 oncogene are among the most prevalent alterations in human malignancies [28]. The cancer-suppressive effect of TP53 is primarily obtained by disrupting the Wnt/β-catenin pathway, thereby preventing transcription factor 4 (TCF4) from binding to the chromatin [29, 30]. Thus, the inactivation of the Wnt/β-catenin/TCF4 pathway is an important step in the inhibition of carcinogenesis by TP53. Multi-tissue tumor studies have shown that TCF4 is highly expressed in human malignancies with aberrant Wnt/β-catenin signaling [31, 32], and that aberrant intracellular β-catenin accumulation and nuclear translocation can also enhance TCF4 transactivation [33–35]. Aberrant activation of TCF4 further enhances its binding to target gene E-box sequences, activating target genes transcription and promoting tumor proliferation, vascular invasion, and distal metastasis [36]. Consequently, TP53 mutations are frequently employed as potential prognostic and forecasting markers, as well as intervention targets for oncology therapeutics. Oridonin has been implicated in the regulation of TP53 expression in tumor cells [37–39], but the precise mechanisms and implications are unknown.

The objective of this research was to examine the relationship between the carcinogenic impact of oridonin and Endoplasmic Reticulum stress and its associated biological mechanisms. Our results showed that oridonin activated TP53 expression in colorectal cancer cells and inhibited TCF4. The inhibition of TCF4 directly caused Endoplasmic Reticulum stress dysregulation and promoted tumor cell death.

## Material and methods

### Reagents

**Fig. 1:**
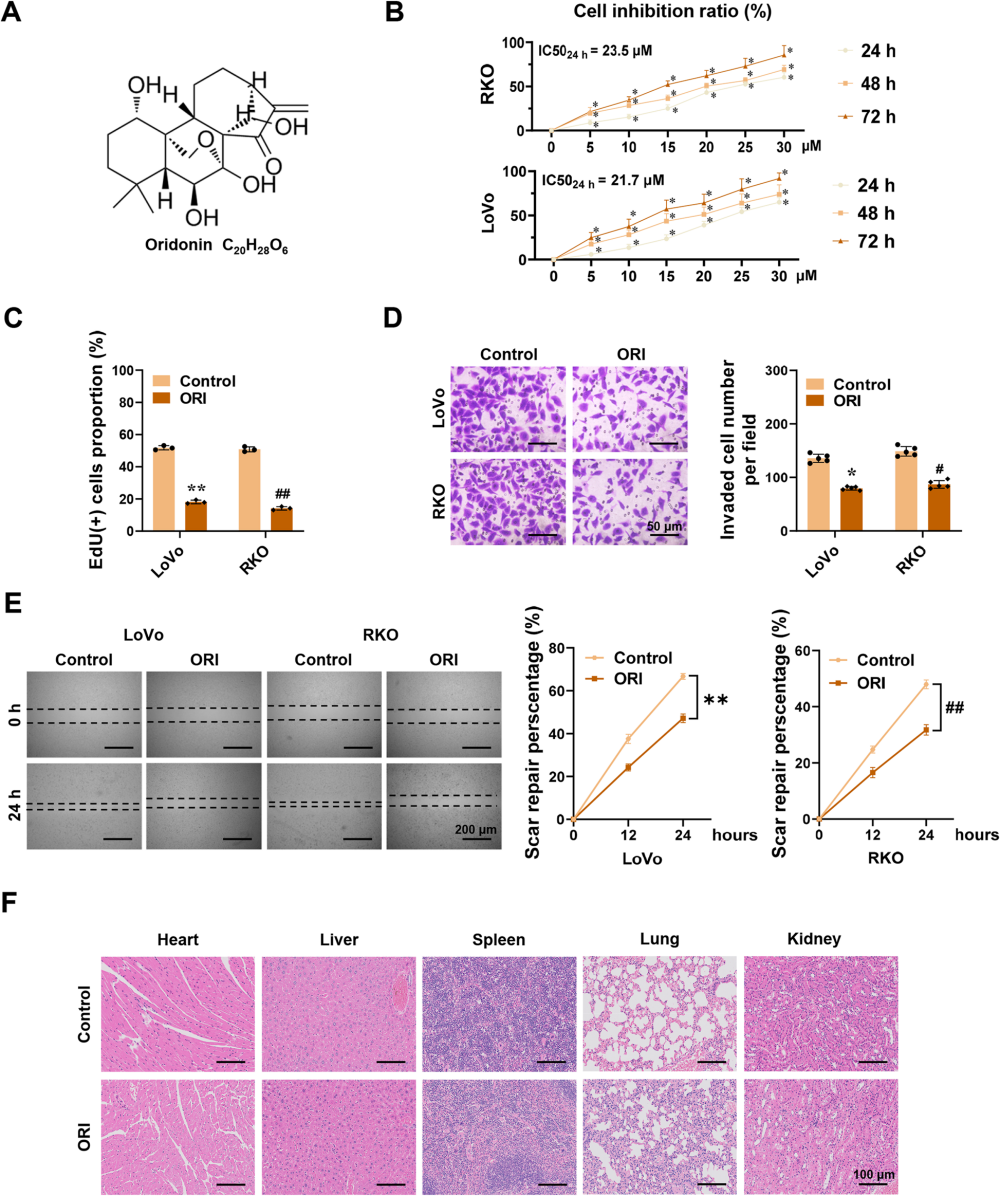
Oridonin prevents the development of colorectal cancer. **A** Molecular structure of oridonin. **B** Cell viability was measured by CCK8. RKO and LoVo cells were treated with oridonin (0, 5, 10, 15, 20, 25, 30 μM) for 24, 48, or 72 h. The IC50 of oridonin for 24 h was marked in the right panel (*n* = 6; IC50_LoVo_ = 21.7 μM, IC50_RKO_ = 23.5 μM). **C-E** EdU staining, Tranwell, and Wound healing assays indicate the proliferation, invasion, and migration of LoVo and RKO cells after treatment with or without 22 μM oridonin for 24 h (*n* = 3). Scale bars of D: 50 μm. Scale bars of E: 200 μm. **F** HE staining of the hearts, livers, spleens, lungs, and kidneys of tumor-bearing athymic nude mice treated with oridonin (ORI, 160 mg/kg) or DMSO (*n* = 5). Scale bars: 100 μm. The statistical results were presented as mean ± SD. Student’s t-test compared the difference in C and D; two-way ANOVA compared the difference in B and E. * *P* < 0.05, ** *P* < 0.01 compared with the Control in LoVo cells; # *P* < 0.05, ## *P* < 0.01 compared with the Control in RKO cells; * *P* < 0.05 compared with the oridonin (0 μM) in B. ORI: oridonin; CCK8: cell counting kit-8; IC50: the half maximal inhibitory concentration; EdU: 5-Ethynyl-2'-deoxyuridine; HE staining: hematoxylin–eosin staining; DMSO: dimethyl sulfoxide; SD: standard deviation; ANOVA: analysis of variance

Oridonin (C_20_H_28_O_6_, CAS No. 28957–04-2, purity: 98%) was purchased from Yuanye Bio-Technology Co. (Shanghai, China). Oridonin was dissolved in dimethyl sulfoxide (DMSO, Sigma, D2650) and kept at 4 °C. In Fig. 1A, the chemical structure is depicted. LF3 (CAS: 664969–54-4) was purchased from Selleck (https://www.selleck.cn/). Z-VAD-FMK (Z-VAD, HY-16658B), Chloroquine (CQ, HY-17589A), and 4-Phenylbutyric acid (4-PBA, HY-A0281) were purchased from MCE (https://www.medchemexpress.cn/). Primary antibodies: ATF4 (Proteintech, 10,835–1-AP), CHOP (Proteintech, 15,204–1-AP), TCF4 (Proteintech, 22,337–1-AP), CFTR (Proteintech, 20,738–1-AP), FYN (Proteintech, 66,606–1-Ig), YOD1 (ABclonal, A13270), IRE1α (Cell Signaling Technology, Cat# 3294), p-IRE1α (Novus Biologicals, NB100-2323), PERK (Proteintech, 24,390–1-AP), p-PERK (ABclonal, AP0886), TP53 (Proteintech, 60,283–2-Ig), Wnt (Abcam, Ab15251), β-catenin (Abcam, Ab16051), β-actin (Proteintech, 66,009–1-Ig). Secondary antibodies: HRP enzyme-labeled goat anti-rabbit IgG (Servicebio, GB23303) and HRP enzyme-labeled goat anti-mouse IgG (Servicebio, GB23301).

### Cell lines and cell culture

The human cell lines including NCM460 (BFN608006385) purchased from Shanghai Cell Bank, RKO (CRL-2577), LoVo (CCL-229), HCT116 (CCL-247), SW480 (CCL-228), HeLa (CCL-2), PC-3 (CRL-1435), and A549 (CCL-185) were purchased from the American Type Culture Collection (ATCC, Rockville, MD). RKO was cultured in Eagle’s minimum essential medium (MEM) (Gibco BRL, Grand Island, NY) supplemented with 10% FBS (Gibco, USA) and 1% antibiotics (100 U/mL penicillin, 100 μg/mL streptomycin). NCM460, LoVo, HCT116, SW480, HeLa, PC-3, and A549 were cultured in DMEM (High Glucose) Medium (Gibco, Carlsbad, CA, USA) supplemented with 10% FBS (Gibco, USA) and 1% antibiotics (100 U/mL penicillin, 100 μg/mL streptomycin). The cells were kept at 37 °C in an incubator containing 5% CO_2_.

### Plasmids and transfection

Human TCF4 eukaryotic expression plasmid (pCMV-mCherry-TCF4-Puro), RNA interference plasmid (pLV3-U6-mCherry-sh-TCF4 #1, #2 and pLV3-U6-mCherry-sg-TP53-Puro), overexpression control plasmid (pCMV-Mock), knockdown control plasmid (pLV3-U6-sh-Scb and pLV3-U6-sg-Scb) were purchased from Shanghai GeneChem Technology Company.

When the cells were cultured in a 6-well plate until the density reached about 80%, the cell medium was replaced 4 h before the transfection, and then the plasmid-transfection reagent suspension was added to the medium, and then the cells were returned to the constant temperature incubator. After one day, the fluorescence expression in the cells could be observed. Subsequently, puromycin or neomycin screening was performed to obtain TCF4 stable overexpression cell line (TCF4), TCF4 knockdown cell lines (sh-TCF4 #1 and sh-TCF4 #2), TP53 knockdown cell lines (sg-TP53) and corresponding blank control cell lines (Mock, sh-Scb, and sg-Scb). And they will be employed in the following research.

### Cell Counting Kit-8 cell viability assay

To detect the cell viability, cells were plated in 96-well plates (0.5 × 10^4^ cells/well) treated with 200 μl medium. Then, various concentrations of oridonin were added. 6 replications were established for each group. After 24, 48, and 72 h, 10 μl of the Cell Counting Kit-8 (CCK8, Kum-amoto, Japan) solution was added to each well and maintained in the dark for 2 h. The absorbance value of all wells was measured using a microplate reader (Tecan Infinite F50, Switzerland) at 450 nm. The cell survival rate was calculated according to the following formula: Cell Viability Rate (%) = (A_EX_—A_OE_)/(A_NC_—A_OE_) × 100%; Cell Inhibition Rate (%) = 1-Cell Viability Rate (%).

### 5-Ethynyl-2'-deoxyuridine (EdU) proliferation assay

Cell proliferation ability was assessed by BeyoClickTM Edu-594 Cell Proliferation Assay Kit (Beyotime, C0078S) following the guidelines. RKO and LoVo cells were planted at a density of 4 × 10^3^ cells per well in 24-well plates. After 24 h of oridonin treatment, the cells were incubated with 10 μM EdU (Beyotime, China) for two hours. Next, the cells were fixed using Immunol Staining Fix Solution (Beyotime, P0098) for 15 min at room temperature. After washing three times with PBS containing 3% BSA, the cells were incubated for 15 min at room temperature in PBS containing 0.3% Triton X-100. The cells were then treated with Click-iT^®^ reaction cocktails at room temperature and out of the dark for 30 min. The fluorescence images were photographed under a fluorescence microscope (Olympus, Japan) after the nuclei were stained by Hoechst 33,342 for 10 min at room temperature. Image J software was utilized to evaluate the percentage of EdU-positive cells.

### Wound healing assay

Cell migration ability was determined by Wound healing assays. Cells were cultured at a 6-well plate until they were distributed equally and the density reached approximately 95%. Then, 200 μl of sterile pipette tip was used to scratch lines, ensuring that each scratch had the same width. PBS washed three times, cells were cultured in a serum-free medium with DMSO or oridonin at 37 ℃ of 5% CO_2_. The cell migration was observed and imaged at 0, 12, and 24 h by using a fluorescent microscope (Olympus, Japan). Image J was used to calculate the distances of the scratch.

### Transwell assay

Twenty-four-well migration chambers with 8-μm pore membrane (Corning, USA) were used to assess cellular invasion. Then, Matrigel (Corning, USA) covered it. Briefly, the lower chambers were added with 500 μl fresh medium containing 10% FBS to attract the cells. Next, 200 μl of a serum-free cell suspension containing 3 × 10^3^/ml of cells was added to the Matrigel-coated upper chambers. These chambers were incubated in 37 ℃ of 5% CO_2_ for 24 h, then the invading cells were fixed with methanol for 15 min, stained with crystal violet for 2 h, and photographed under a fluorescence microscope (Olympus, Japan). The number of invasive cells in each well was determined by observing and photographing five random fields in each well. The five random fields were determined by drawing equidistant lines, and there were three technical replicate holes in each experiment. The mean of the counts of the three wells was taken as the result of one biological experiment. The experimental procedure was repeated three times for biological replicates.

### Reverse Transcription quantitative Polymerase Chain Reaction (qRT-PCR)

According to the protocol, total RNA was extracted using the RNA isolation Total RNA Extraction Reagent (Vazyme, Nanjing, R401-01). And cDNA was prepared after reverse transcription using HiScript^®^ II qRT SuperMix. qRT-PCR was performed according to the instruction (ChamQTM SYBR^®^ qPCR Master Mix) in StepOne Plus (Applied Biosystems). The primers were synthesized by Tsingke Biology (Wuhan, China), and β-actin was used as the reference gene. The sequences of all primers were listed in Table 1.

**Table 1:** Sequences of primers for qRT-PCR

Gene	Primers	Sequence	Product size (bp)
TCF4	Forward Reverse	5′-ACTGCCGACTACAATAGG-3′ 5′-TGGATAGCTCAAACGTTC-3′	228
NFIC	Forward Reverse	5′-CCCTACGACGTCCATCCTAC-3′ 5′-TTGAGCTGACCACTTCCATT-3′	168
STAT3	Forward Reverse	5′-CCGTGGAACCATACACAAAG-3′ 5′-CTAAAGTGCGGGGGGACATC-3′	288
CFTR	Forward Reverse	5′-CATTTACGTGGGAGTAGC-3′ 5′-ACAAAGATGTAGGGTTGT-3′	300
FYN	Forward Reverse	5′-CTACAACAACTTCCACGC-3′ 5′-GCTCGGAAGGAGATTGGT-3′	212
YOD1	Forward Reverse	5′-CTTGGGGAGGAGCAATAGAGAT-3′ 5′-GAGAAAATGGTCAGAGGAGGTG-3′	208
TP53	Forward Reverse	5′-CCGGACGATATTGAACAATG-3′ 5′-CAAGAAGCCCAGACGGAAAC-3′	204
β-actin	Forward Reverse	5′-TGCCCATCTACGAGGGGTATG-3′ 5′-TCTCCTTAATGTCACGCACGATTT-3′	156

### Western blot

Proteins were extracted from cells using RIPA buffer (Beyotime, China) and total protein concentrations were detected using a BCA protein assay kit (Beyotime, China) according to the protocol. Equal amounts (30 μg) of total protein were separated by 12% SDS-PAGE and then transferred to 0.45 μm PVDF membranes. Then, membranes were blocked with 5% skim milk for 1 h and incubated overnight at 4 °C with the primary antibodies. After washing three times with TBST, membranes were incubated with the secondary antibodies for 1 h at room temperature. Proteins were detected by a UVP Bio-Spectrum Imaging System (BioSpectrum 600) and analyzed using Image J software. The protein expression levels were normalized by β-actin.

### Cell immunofluorescence

To investigate the expression level of ATF4 and CHOP in cells, cells were seeded on coverslips, fixed with methanol for 15 min, and then incubated with anti-CHOP primary antibody (1:400), or anti-ATF4 primary antibody (1:50) at 4 °C overnight. The cells were washed three times with PBS and then incubated with the secondary antibody: Goat Anti-Rabbit IgG H&L (Alexa Fluor^®^ 594) (Abcam, ab150080). And the nuclei were stained with DAPI (Beyotime, #C1005). Finally, the cells were photographed and analyzed using a Nikon A1Si Laser Scanning Confocal Microscope (Nikon Instruments Inc., Japan).

### Endoplasmic reticulum tracker assay

According to the manufacturer's instructions, the Endoplasmic Reticulum tracker (ER tracker-red, Beyotime, #C1041) was used for ER localization. Briefly, cells were incubated for 30 min at 37 °C of 5% CO_2_ in a pre-warmed ER tracker dye working solution. The cells were washed with PBS and fixed with 4% paraformaldehyde. And the nuclei were stained for 10 min at room temperature with Hoechst 33,342 (Beyotime, China). Then cells were observed and photographed under a Nikon A1Si Laser Scanning Confocal Microscope (Nikon Instruments Inc., Japan).

### Determination of Reactive Oxygen Species (ROS)

Intracellular ROS levels were detected using the ROS Assay Kit. In brief, the cells were incubated with 10 μM DCFH–DA (Beyotime, China) for 20 min at 37 ℃. Then, the cells were washed three times with PBS and stained with Hoechst 33,342 (Beyotime, China) for 10 min at room temperature. The DCF fluorescence intensity was then observed and photographed under a Nikon A1Si Laser Scanning Confocal Microscope (Nikon Instruments Inc., Japan). In addition, the ROS level was also measured using DCFH–DA by a flow cytometer (FCM, Beckman Coulter, USA) at 488 nm excitation and 525 nm emission, and the data were analyzed using FlowJo 8.1 software.

### Ca^2+^ concentration detection

To detect intracellular free Ca^2+^ levels, Fluo-3 AM (S1056, Beyotime, China) was used. In brief, the cells were incubated with Fluo-3 AM for 1 h at 37 ℃. Then, the cells were washed with PBS three times, and the absorbance value of all wells was measured using a microplate reader (Tecan Infinite F50, Switzerland) at 488 nm excitation and 530 nm emission. Ca^2+^ concentrations were obtained by substituting the fluorescence values into the standard curve. And each group was set up with at least six independent replicate experiments.

### In vivo tumorigenesis assays

All animal studies were performed following the NIH Guide for the Care and Use of Laboratory Animals and were approved by the Animal Care Committee of Tongji Medical College (approval number: SYXK2021-0057). 4-week-old male BALB/c-nude mice (Hubei BIONT Biotechnology Co.) were bred in specific pathogen-free conditions. For in vivo tumor growth studies, 2 × 10^6^ cells (suspended in 0.2 ml phosphate-buffered saline) were subcutaneously injected into the right flank after 1 week of feeding. Two weeks following the injection of tumor cells, oridonin gavage (160 mg/kg) was administered for 14 days. Mice were recorded for tumor volume and imaged using In-Vivo Xtreme II small animal imaging system (Bruker Corporation, Billerica, MA). At the end of the experiment, the mice were sacrificed, and the tumor samples were collected for weighing and immunohistochemistry studies.

### Hematoxylin–eosin staining

Immediately after the nude mice were sacrificed, the hearts, livers, spleens, lungs, and kidneys were removed and fixed in 10% neutral buffered formalin at 4 °C. Serial tissue sections at 4 µm were stained with hematoxylin and eosin and examined for pathological changes under a fluorescence microscope (Olympus, Japan).

### Immunohistochemistry

The tumor tissues were fixed, paraffin-embedded, and sectioned (4 μm). After deparaffinization and rehydration, the sections were boiled for 15 min in citrate sodium solution to recover antigens and then submerged for 10 min in 3% H_2_O_2_ to quench endogenous peroxidase activity. The sections were then blocked with 10% serum for 1 h at 37 °C. The primary antibodies (MKI67/Ki-67: Proteintech, 27,309–1-AP; PECAM1/CD31: Proteintech, 66,065–2-Ig) were added and incubated overnight at 4 °C. After washing, the sections were coated with HRP-conjugated second antibody and then incubated at 37 °C for 1 h. The subsequent procedures were performed according to the manufacturer’s instructions.

### Data availability

Endoplasmic Reticulum stress gene sets were obtained from AmiGO 2 (http://amigo.geneontology.org/amigo/). Potential transcription factors regulating gene expression were screened by applying for PWMs software [40]. Public datasets on the correlation between gene expression and survival in cancer patients were obtained from the GEO database (https://www.ncbi.nlm.nih.gov/geo/, accession numbers: GSE14333, GSE17538, and GSE33114).

### Statistical analysis

All data were expressed as mean ± standard deviation (SD) and analyzed by GraphPad Prism 8.0. Survival curves were estimated using the Kaplan–Meier method with a log-rank test. Correlation analysis was conducted by GraphPad Prism 8.0 with Pearson test. Student’s t-test was used to detect between-group differences. One-way or two-way analysis of variance (ANOVA) was used for comparisons of multiple groups. *P* < 0.05 was defined as statistically significant. Significant difference is expressed as *, # *P* < 0.05 or **, ## *P* < 0.01.

## Results

### Oridonin prevents the development of colorectal cancer

To assess the cytotoxic and inhibitory effects of oridonin on colorectal cancer (CRC) cells, the CCK-8 assay was used. LoVo and RKO cells were treated for 24, 48, and 72 h with the specified concentrations of oridonin (0, 5, 10, 15, 20, 25, and 30 µM). The results showed that oridonin inhibited the growth of CRC cells in a time- and dose-dependent manner (Fig. 1B). The half maximal inhibitory concentration (IC_50_) of oridonin in LoVo and RKO cells at 24 h was 21.7 µM and 23.5 µM respectively. In anticipation of a more pronounced effect, we selected a concentration of 22 µM oridonin for subsequent experiments. After 24 h of treatment with oridonin, the proportion of EdU-positive cells in both RKO and LoVo cells was significantly reduced in the treatment group compared with the control group (Fig. 1C). To investigate the impact of oridonin on the metastatic and invasion potential of CRC cells, wound healing and invasion assays were performed. As shown in Figs. 1D and E, compared to the control, oridonin significantly reduced the migration and invasion of CRC cells. To assess the side effects of oridonin in vivo, five-week-old athymic nude mice were gavaged continuously with oridonin for a fortnight after the subcutaneous xenograft modeling. After the mice were sacrificed, organs were collected and stained for hematoxylin–eosin. No significant histological were observed in the hearts, livers, spleens, lungs, and kidneys between the oridonin and control group, indicating that this concentration is not significantly toxic to the organs of tumor-bearing nude mice (Fig. 1F).

### Oridonin induces endoplasmic reticulum stress in colorectal cancer

**Fig. 2:**
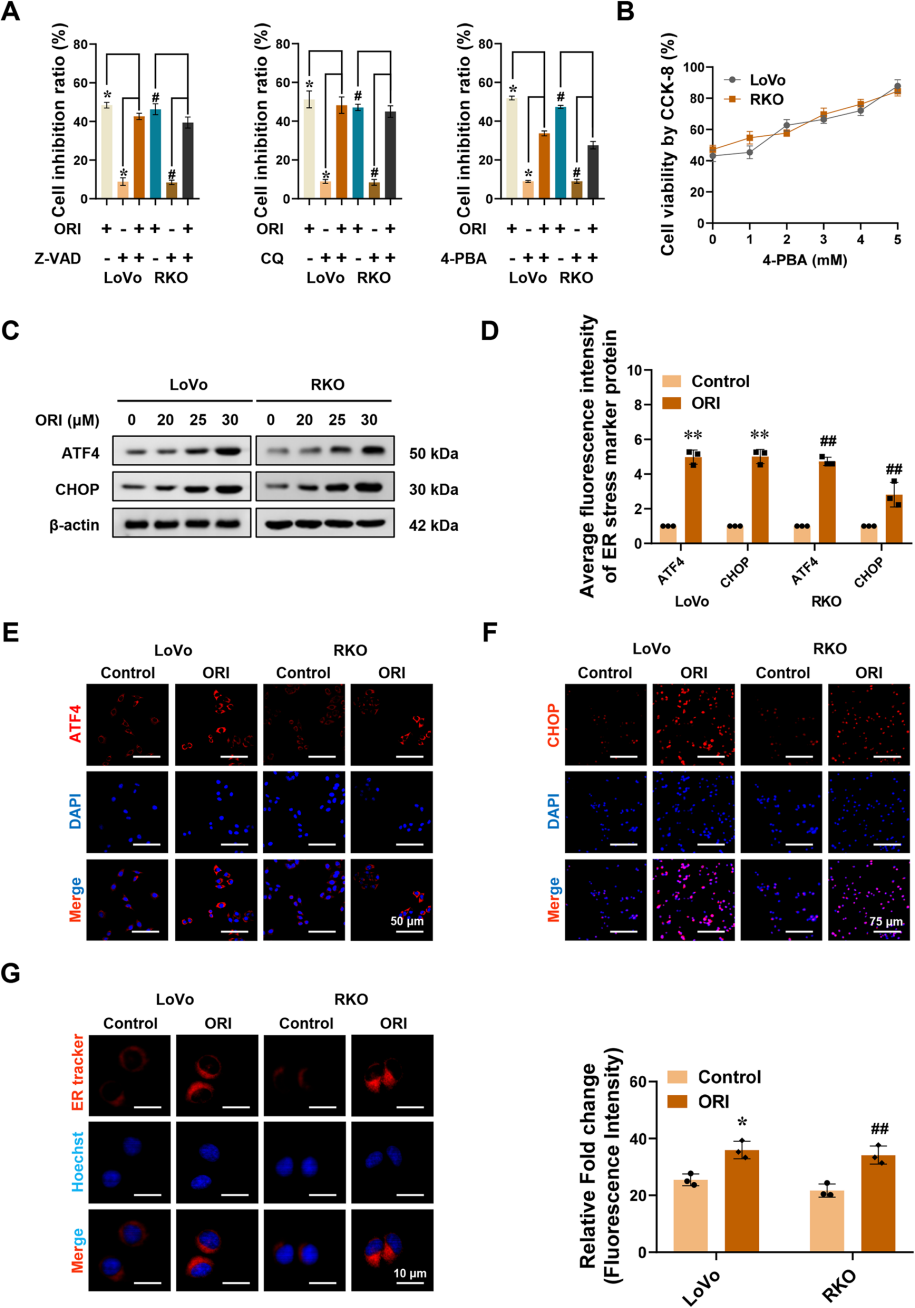
Oridonin induces Endoplasmic Reticulum stress in colorectal cancer. **A** CCK-8 assay showing the cell inhibition ratio of LoVo and RKO cells. LoVo and RKO cells were treated with oridonin (ORI, 22 μM) and with or without Z-VAD (10 μM) or CQ (20 μM) or 4-PBA (3 mM) for 24 h (*n* = 6). **B** CCK-8 assay showing the cell viability of LoVo and RKO cells treated with oridonin (22 μM) and 4-PBA (0, 1, 2, 3, 4, 5 mM) for 24 h (*n* = 6). **C** Western blot assay showing the expression of the ATF4 and CHOP in LoVo and RKO cells treated with oridonin (0, 20, 25, 30 μM) for 24 h (*n* = 3). **D-F** The quantitative histogram (**D**) and representative fluorescence images (**E**–**F**) for ATF4 and CHOP in LoVo and RKO cells treated with or without oridonin for 24 h. Scale bars of **E**: 50 μm. Scale bars of F: 75 μm (*n* = 3). **G** The representative fluorescence images (left panel) and fluorescence intensity quantitative histogram (right panel) of ER tracker-red in LoVo and RKO cells (*n* = 3). Scale bars: 10 μm. The statistical results were presented as mean ± SD. Student’s t-test compared the difference in **A**, **D**, and **G**; one-way ANOVA compared the difference in **B**. * *P* < 0.05, ** *P* < 0.01 compared with the Control in LoVo cells; # *P* < 0.05, ## *P* < 0.01 compared with the Control in RKO cells. ER: Endoplasmic reticulum; Z-VAD: Z-VAD-FMK; CQ: chloroquine; 4-PBA: 4-Phenylbutyric acid; ATF4: Activating transcription factor 4; CHOP: DNA damage-inducible transcript 3

Our study showed that oridonin could effectively inhibit the proliferation, invasion, and migration of CRC cells in a dose- and time-dependent manner. To further verify whether these putative forms of cell death are associated with the anticancer activity of oridonin in CRC cells. We used various inhibitors of cell death, including Z-VAD-FMK (Z-VAD, a pan-caspase inhibitor), Chloroquine (CQ, an autophagy inhibitor), and 4-Phenylbutyric acid (4-PBA, an ER stress inhibitor). As shown in Fig. 2A, 4-PBA significantly restored cell viability, whereas we noted that Z-VAD and CQ only slightly rescued cell viability in LoVo and RKO cells cultured with oridonin for 24 h. To further explore the association between the anticancer activity of oridonin and ER stress, colorectal cancer cells co-cultured with oridonin were rescued with different concentrations of 4-PBA. We found that the viability of tumor cells increased with increasing 4-PBA concentration, indicating that ER stress is required for oridonin-induced apoptosis (Fig. 2B). Therefore, we hypothesized that ER stress might be a crucial factor in the suppression of colorectal cancer by oridonin.

Activating transcription factor 4 (ATF4) and DNA damage-inducible transcript 3 (DDIT3/CHOP), as key molecules in the classical pathway of Endoplasmic Reticulum stress-induced apoptosis, are used as marker proteins of ER stress [41]. Western blot and immunofluorescence assays showed that the protein expression levels of ATF4 and CHOP were significantly increased in the cells treated with oridonin (Fig. 2C-F). In addition to the changes in marker proteins, ER stress can also cause changes in the morphology of ER. After co-cultured with oridonin for 24 h, the fluorescence intensity of ER tracker-red in LoVo and RKO cells increased significantly, and the Endoplasmic Reticulum expanded, swollen, and vacuolated (Fig. 2G-H). These findings support the hypothesis that the anticancer effects of oridonin are associated with ER stress.

### TCF4 functions as an essential transcription factor in the regulation of ER stress

**Fig. 3:**
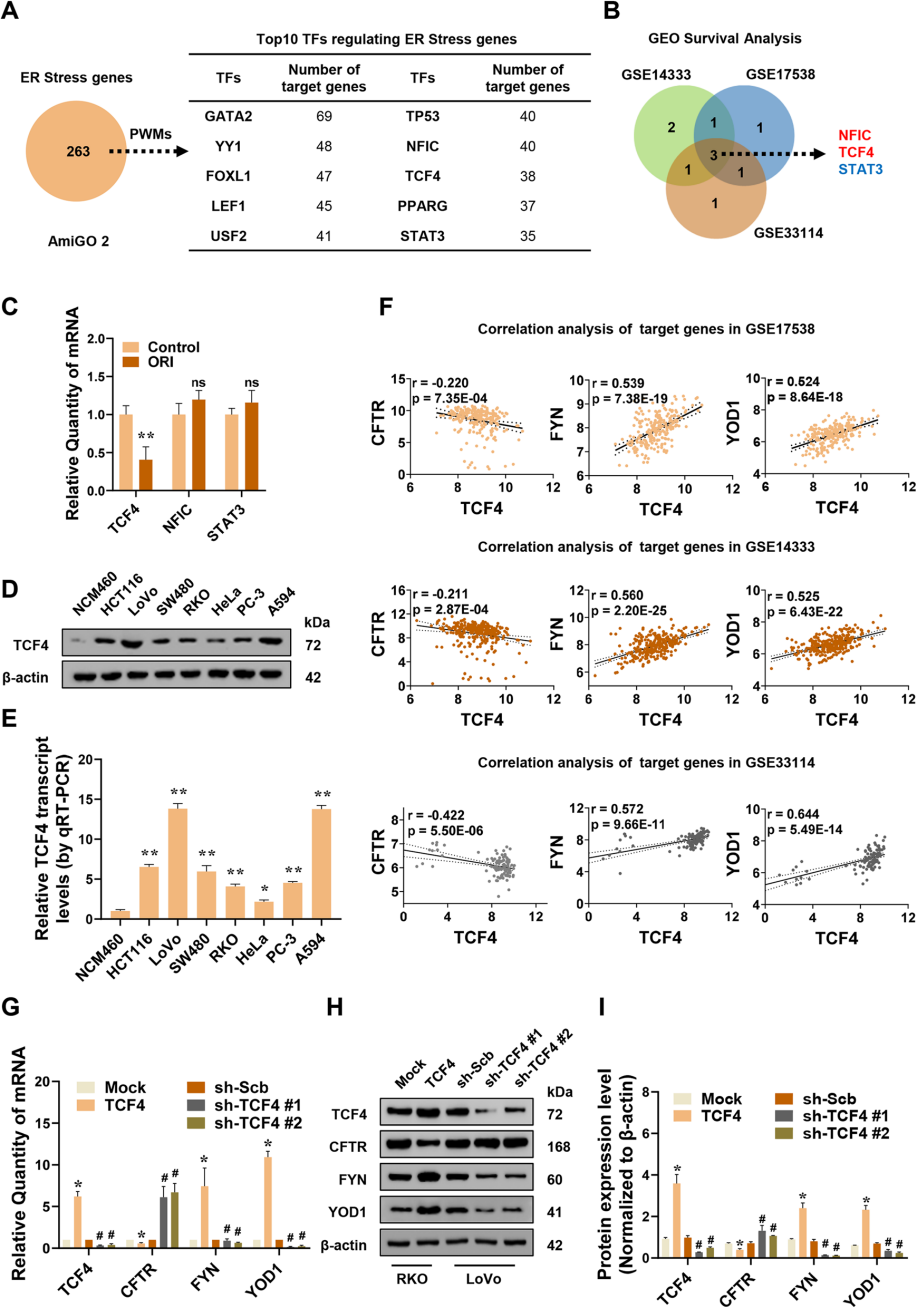
TCF4 functions as an essential transcription factor in the regulation of ER stress. **A** The top 10 transcription factors (right table) regulating the expression of ER stress genes from the AmiGO 2 database (left panel) were analyzed by PWMs software. **B** Kaplan–Meier survival analysis in colorectal cancer GEO database (GSE14333, GSE17538, GSE33114) finding three transcription factors regulating ER stress genes. **C** qRT-PCR assay revealing the levels of TCF4, NFIC, and STAT3 in RKO cells treated with or without oridonin (*n* = 3). Normalized to β-actin. **D-E** Western blot and qRT-PCR assays showing the levels of TCF4 in different cell lines (*n* = 3). Normalized to β-actin. **F** Correlation analysis of TCF4 and target genes in colorectal cancer GEO database. **G-I** Western blot and qRT-PCR assays revealing the expression of TCF4, CFTR, FYN, YOD1 in RKO or LoVo cells stably transfected with empty vector pCMV-Mock (Mock), TCF4 eukaryotic expression plasmid pCMV-TCF4 (TCF4) or knockdown control plasmid pLV3-U6-sh-Scb (sh-Scb), RNA interference plasmid pLV3-U6-shTCF4 #1 (sh-TCF4 #1) and pLV3-U6-shTCF4 #2 (sh-TCF4 #2) (*n* = 3). Normalized to β-actin. The statistical results were presented as mean ± SD. Student’s t-test compared the difference in C-E and G-I; Linear regression analysis in **F**. * *P* < 0.05, ** *P* < 0.01 compared with Control, NCM460, Mock in **C**, **D**, **E**, **G**, and **I**; # *P* < 0.05 compared with sh-Scb in **G** and **I**; *ns* compared with Control in **C**. TFs: transcription factors; PWMs: Position Weight Matrices; GEO: Gene Expression Omnibus database; qRT-PCR: reverse transcription-quantitative polymerase chain reaction; TCF4: transcription factor 4; NFIC: nuclear factor 1 C-type; STAT3: signal transducer and activator of transcription 3; CFTR: Cystic fibrosis transmembrane conductance regulator; FYN: Tyrosine-protein kinase Fyn; YOD1: YOD1 deubiquitinase; *ns*: not statistically significant

To investigate the essential transcription factors regulating the expression of ER stress genes, the AmiGO 2 database was utilized to obtain an ER stress-associated gene set. Position Weight Matrices (PWMs) analysis was conducted to predict the transcription factors affecting the expression of these genes, and the top 10 transcription factors were provided based on the number of target genes ranked (Fig. 3A). Among them, 3 were consistently associated with Disease Free Survival (DFS) in CRC patients (Fig. 3B). To investigate the effects of oridonin on the expression of the above three transcription factors, the qRT-PCR assay was conducted. As shown in Fig. 3C, oridonin treatment decreased TCF4 transcription levels compared with the control group but had little effect on Nuclear factor 1 C-type (NFIC) and Signal transducer and activator of transcription 3 (STAT3). Furthermore, according to the detection and analysis of multiple tumor cell lines, the protein and mRNA expression levels of TCF4 were higher in colorectal cancer HCT116, LoVo, SW480, and RKO cell lines than NCM460, a normal intestinal epithelial immortalized cell line. It is also highly expressed in cervical cancer cell line HeLa, prostate cancer cell line PC-3, and non-small cell lung cancer cell line A594 (Fig. 3D-E).

GEO survival analysis was conducted to screen TCF4 target genes associated with the survival of colorectal cancer patients, among which Cystic fibrosis transmembrane conductance regulator (CFTR), Tyrosine-protein kinase Fyn (FYN), and YOD1 deubiquitinase (YOD1) were considered to be eligible (Fig. 3F, Table S1). Subsequently, the expression levels of CFTR, FYN, and YOD1 were detected by Western blot and qRT-PCR in TCF4 overexpression or knockdown cells. Overexpression of TCF4 promoted the mRNA and protein synthesis of YOD1 and FYN but decreased the expression of CFTR. When TCF4 was knocked down, the expression of YOD1 and FYN decreased, while CFTR expression levels increased (Fig. 3G-I).

### TCF4 promotes colorectal cancer progression by inhibiting ER stress

**Fig. 4:**
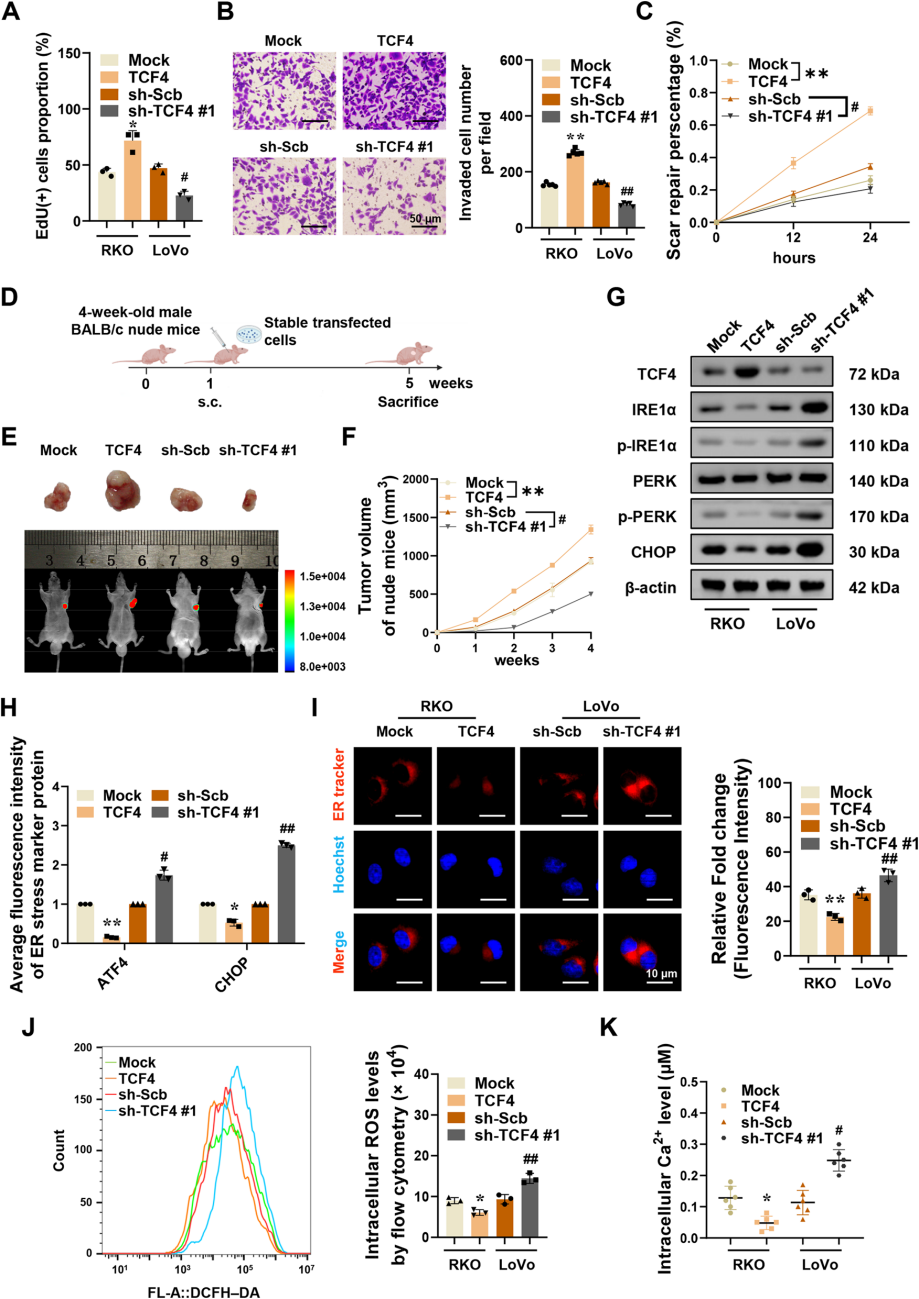
TCF4 promotes tumorigenesis and inhibits ER stress in colorectal cancer. **A-C** EdU staining, Tranwell, and Wound healing assays showing proliferation, invasion, and migration of RKO or LoVo cells stably expressing Mock, TCF4, sh-Scb, or sh-TCF4 #1 (*n* = 3). Scale bars of B: 50 μm. **D** Flowchart of xenografts by subcutaneous injection of RKO cells stably transfected with Mock, TCF4, sh-Scb, or sh-TCF4 #1 into the dorsal flanks of nude mice. **E–F** Representative fluorescence and tumor images (E) and tumor growth curve of xenografts (F) by subcutaneous injection of RKO cells stably transfected with Mock, TCF4, sh-Scb, or sh-TCF4 #1 into the dorsal flanks of nude mice (*n* = 5). **G** Western blot assay showing the expression of TCF4, IRE1α, p-IRE1α, PERK, p-PERK, and CHOP in RKO or LoVo cells stably expressing Mock, TCF4, sh-Scb, or sh-TCF4 #1 (*n* = 3). Normalized to β-actin. **H** The quantitative histogram for the fluorescence images of ATF4 and CHOP in RKO or LoVo cells stably expressing Mock, TCF4, sh-Scb, or sh-TCF4 #1. **I** Representative fluorescence images and the quantitative histogram of ER tracker-red in RKO or LoVo cells stably expressing Mock, TCF4, sh-Scb, or sh-TCF4 #1 (*n* = 3). Scale bars: 10 μm. **J** Flow cytometry analysis of intracellular ROS levels in RKO or LoVo cells stably expressing Mock, TCF4, sh-Scb, or sh-TCF4 #1 (*n* = 3). **K** The calcium indicator Fluo-3 AM detects intracellular Ca^2+^ levels in RKO or LoVo cells stably expressing Mock, TCF4, sh-Scb, or sh-TCF4 #1 (*n* = 6). The statistical results were presented as mean ± SD. Student’s t-test compared the difference in A-B and H–K, and two-way ANOVA compared the difference in C and F. * *P* < 0.05, ** *P* < 0.01 compared with Mock; # *P* < 0.05, ## *P* < 0.01 compared with sh-Scb. IRE1α: endoplasmic reticulum to nucleus signaling 1-alpha; p-IRE1α: phosphor-IRE1α; PERK: eukaryotic translation initiation factor 2 alpha kinase 3, EIF2AK3; p-PERK: phosphor-PERK; ROS: reactive oxygen species; Ca^2+^: calcium

Then, we explored the involvement of ER stress roles of TCF4 in colorectal cancer. The proliferation, migration, and invasion capabilities of RKO or LoVo cells were increased or decreased by stable overexpression or silencing of TCF4 respectively (Fig. 4A-C). Athymic nude mice were utilized to detect TCF4 tumorigenicity in vivo (Fig. 4D). Four weeks after modeling subcutaneous tumor transplantation in nude mice, the mice were sacrificed and the transplanted tumors were excised and weighed (Fig. 4E, and S1A-B). Experimental results showed that colorectal cancer cells stably expressing TCF4 or knocking down TCF4 were subcutaneously injected into athymic nude mice, causing an increase or decrease in xenograft tumor growth (Fig. 4F), weight (Figure S1C), MKI67/Ki-67 (marker of proliferation Ki-67) index and PECAM1/CD31 (platelet and endothelial cell adhesion molecule 1)-positive microvessels (Fig. S1D). In addition, YOD1 and FYN were upregulated in subcutaneous xenograft tumors formed by RKO cells stably overexpressing TCF4 in these athymic nude mice, along with the downregulation of CFTR protein and mRNA levels (Fig. S1E-F). However, in xenografts formed by LoVo cells TCF4 knocking down, CFTR expression is upregulated, but protein and mRNA production of YOD1 and FYN is decreased (Fig. S1E-F). As determined by the results of in vitro and in vivo studies, TCF4 is important for the development of colorectal cancer.

The key protein markers, p-IRE1α, PERK, p-PERK, CHOP, and ATF4, in the classic pathway of ER stress-mediated cell death, were selected as ER stress sensor markers. p-IRE1α expression was inhibited or enhanced in RKO cells stably overexpressing TCF4 or LoVo cells TCF4 knocking down respectively (Fig. 4G). The expression of PERK was not significantly altered, but p-PERK was significantly reduced or increased, and the CHOP protein level decreased or increased (Fig. 4G). The protein expression of ER stress markers ATF4 and CHOP was decreased or increased by stable overexpression or silencing of TCF4 respectively (Fig. 4H, and S1G-H). After stably overexpressing TCF4, the fluorescence intensity of ER tracker-red decreased in RKO cells, and the morphology of the ER shrank. Whereas, the fluorescence intensity of LoVo cells that silenced TCF4 was significantly increased, and the endoplasmic reticulum expanded and vacuolated (Fig. 4I). Reactive Oxygen Species (ROS) generation and Ca^2+^ release from the Endoplasmic Reticulum are key features of ER stress [42–44]. ROS production was reduced in cells with stable expression of TCF4, accompanied by a decrease in intracellular Ca^2+^ concentration. However, LoVo cells with TCF4 knockdown had increased intracellular Ca^2+^ and ROS levels significantly (Fig. 4J-K).

### Oridonin inhibits colorectal cancer though blocking the inhibition of ER stress by TCF4

**Fig. 5:**
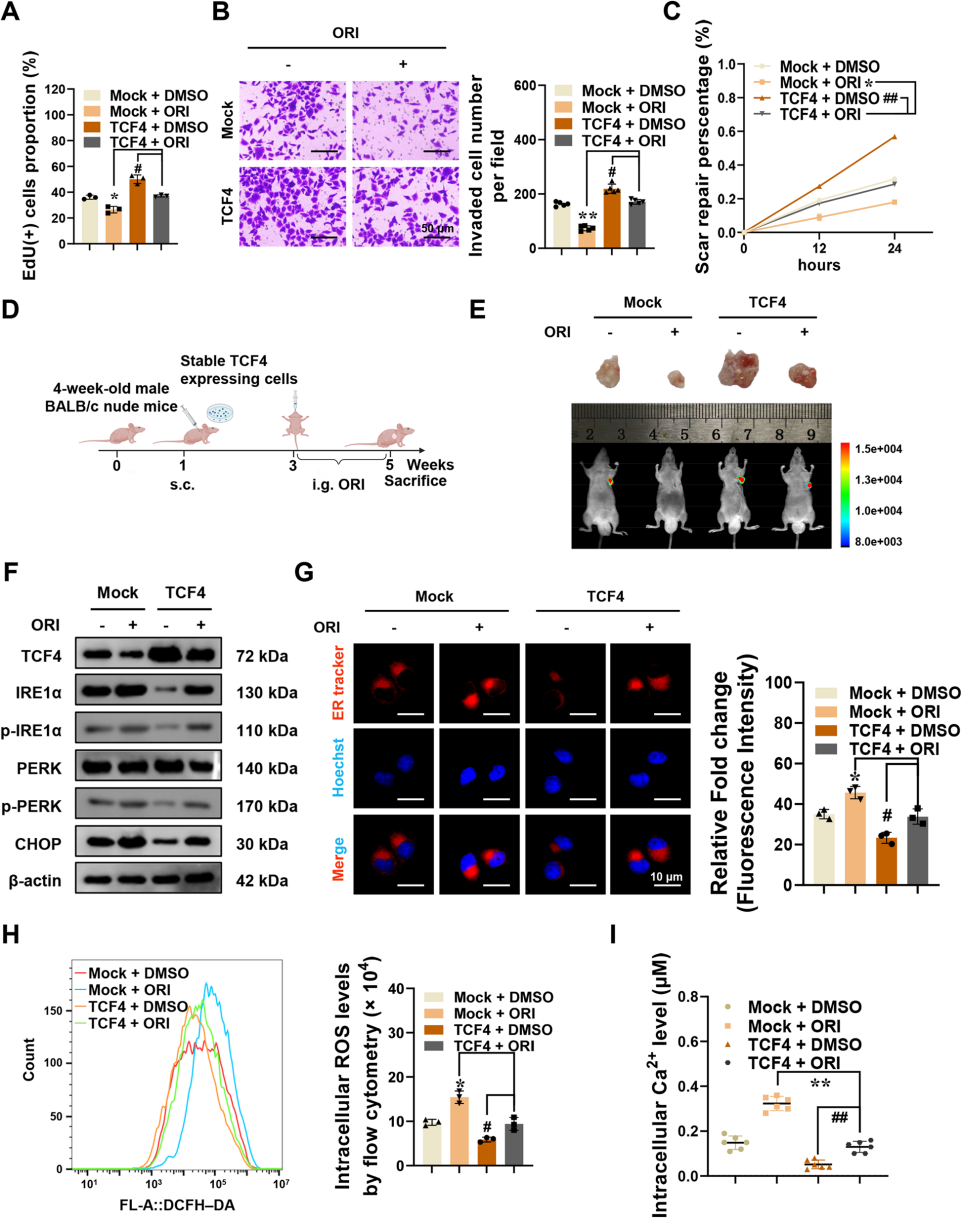
Oridonin blocks the inhibition of ER stress by TCF4 in colorectal cancer. **A-C** EdU, Tranwell, and Wound healing assays showing proliferation, invasion, and migration of RKO cells stably transfected with Mock or TCF4 and treated with or without oridonin (*n* = 3). Scale bars of B: 50 μm. **D** Flowchart of xenografts by subcutaneous injection of RKO cells stably transfected with Mock or TCF4 and treatment with or without oridonin. **E** Representative fluorescence and tumor images of the xenografts by subcutaneous injection of RKO cells stably transfected with Mock or TCF4 and treatment with or without oridonin (*n* = 5). **F** Western blot assay indicating the levels of TCF4, IRE1α, p-IRE1α, PERK, p-PERK, and CHOP in RKO cells stably transfected with Mock or TCF4 and treated with or without oridonin (*n* = 3). Normalized to β-actin. **G** Representative fluorescence images and the quantized histogram of ER tracker-red in RKO cells stably transfected with Mock or TCF4 and treated with or without oridonin (*n* = 3). Scale bars: 10 μm. **H** Flow cytometry analysis of intracellular ROS levels in RKO cells stably transfected with Mock or TCF4 and treated with or without oridonin (*n* = 3). **G** The calcium indicator Fluo-3 AM detects intracellular Ca^2+^ levels in RKO cells stably transfected with Mock or TCF4 and treated with or without oridonin (*n* = 6). The statistical results were presented as mean ± SD. Student’s t-test compared the difference in A-B and G-I, and two-way ANOVA compared the difference in C. * *P* < 0.05, ** *P* < 0.01 compared with Mock + ORI, and # *P* < 0.05, ## *P* < 0.01 compared with TCF4 + DMSO

Bioinformatics data analysis and experimental data showed that TCF4 promoted tumor development by inhibiting endoplasmic reticulum stress. Combined with the high correlation between the tumor suppression of oridonin and endoplasmic reticulum stress, it is essential to investigate whether the ER stress regulation of TCF4 is connected to the tumor-suppressive effects of oridonin. The viability, invasion, and migration ability of RKO cells stably overexpressing TCF4 were decreased after 24 h of co-culture with oridonin (Fig. 5A-C). In addition, the expression level of TCF4 in colorectal cancer cells was inhibited by oridonin, which further affected the transcriptional regulation of downstream ER stress-related target genes by TCF4 (Fig. S2A-B).

To evaluate whether oridonin can effectively inhibit the tumorigenesis of xenograft tumors in vivo, athymic nude mice were subcutaneously injected with TCF4 or Mock cells to establish subcutaneous xenograft tumor models. Two weeks after the injection, part of the tumor-bearing nude mice was gavaged with oridonin for 14 days (Fig. 5D-E). Two weeks after modeling, the tumor volume of subcutaneous xenografts increased, and the xenograft tumors in the TCF4 group grew faster than those in the Mock group (Fig. S2C). However, the growth rate of transplanted tumors in TCF4 or Mock nude mice after oridonin gavage was significantly slowed down (Fig. S2C). And the endpoint weight, fluorescence signal intensity, Ki-67 proliferation index, and CD31-positive microvessel score of the subcutaneous xenografts were all decreased in subcutaneous xenografts of nude mice receiving oridonin treatment (Figures S2D-G). In addition, the protein expression of FYN and YOD1 decreased after oridonin gavage in TCF4-overexpressed xenograft tumors, while CFTR increased. It showed that oridonin could also regulate the expression of TCF4 in vivo, and thus affect the regulation of ER stress-related genes by TCF4 (Fig. S2H-I).

In vitro, the expression of ER stress marker proteins was increased after treatment with oridonin, which was decreased by overexpression of TCF4 (Fig. 5F). Furthermore, ER tracker-red staining showed that oridonin treatment could significantly reduce the fluorescence intensity in TCF4 overexpressed RKO cells, and the morphology of the endoplasmic reticulum was relatively more regular after oridonin treatment (Fig. 5G). Similar results were obtained in the detections of ROS and Ca^2+^ levels. ROS and Ca^2+^ levels increased after treatment with oridonin in both the Mock and TCF4 group (Fig. 5H-I). Taken together, these results suggested that oridonin decreases the tumorigenesis and aggressiveness of colorectal cancer cells by declining the inhibition of TCF4 on ER stress.

### Oridonin attenuates ER stress by TP53/TCF4 axis in colorectal cancer

**Fig. 6:**
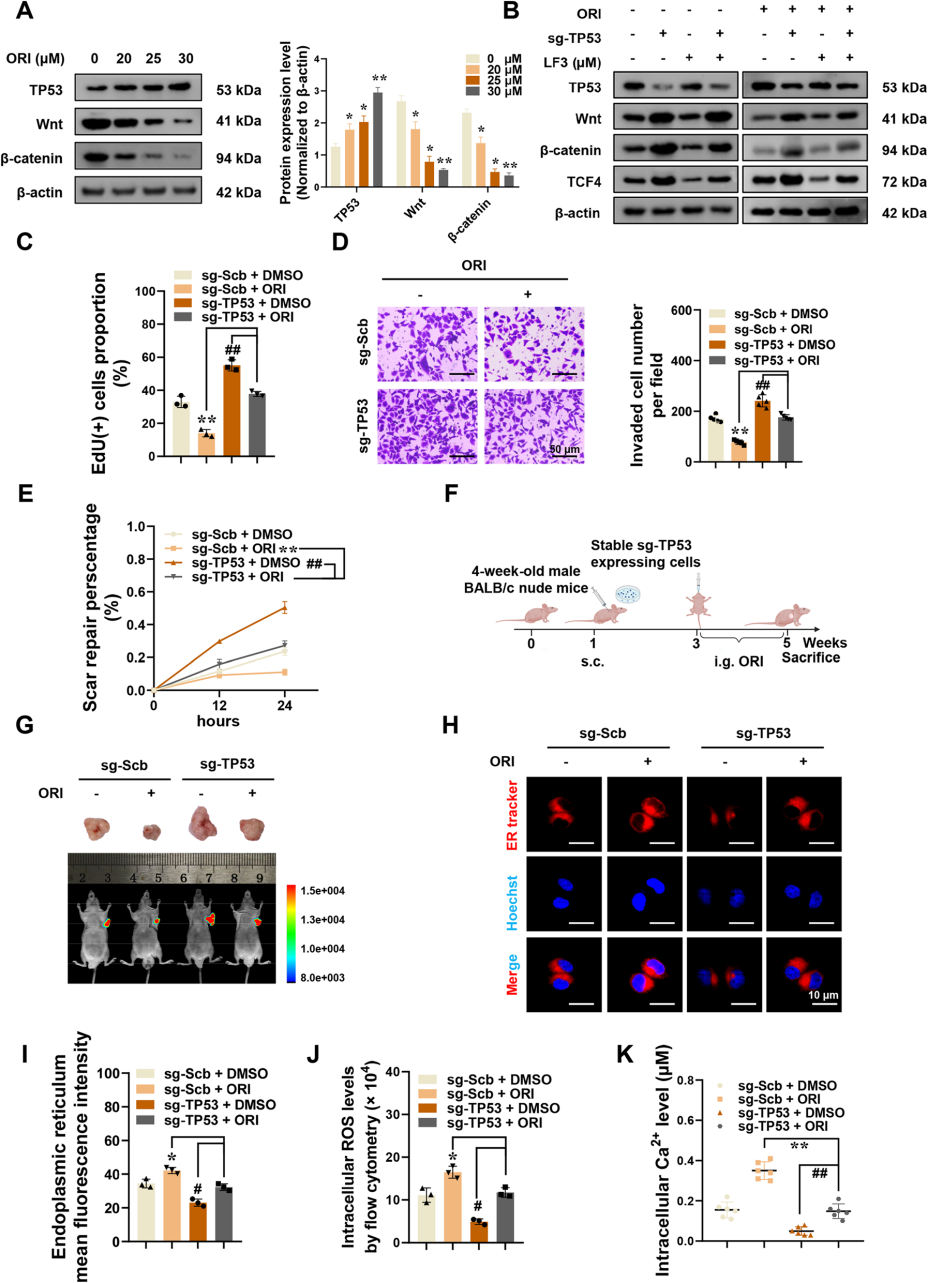
Oridonin attenuates ER stress by TP53/TCF4 axis in colorectal cancer. **A** Western blot assay showing the protein levels of TP53, Wnt, and β-catenin in colorectal cancer cells co-cultured with oridonin (0, 20, 25, 30 μM) for 24 h (*n* = 3). Normalized to β-actin. **B** Western blot assay showing the protein levels of TP53, Wnt, β-catenin, and TCF4 in RKO cells stably transfected with sg-Scb or sg-TP53 and treated with oridonin or LF3 (10 μM) (*n* = 3). **C-E** EdU staining, Tranwell, and Wound healing assays showing proliferation, invasion, and migration of RKO cells stably transfected with sg-Scb or sg-TP53 and treated with or without oridonin (*n* = 3). Scale bars of **D**: 50 μm. **F** Flowchart of xenografts by subcutaneous injection of RKO cells stably transfected with sg-Scb or sg-TP53 and treatment with or without oridonin.** G** Representative fluorescence and tumor images of xenografts by subcutaneous injection of RKO cells stably transfected with sg-Scb or sg-TP53 and treatment with or without oridonin (*n* = 5). **H-I** Representative fluorescence images (**H**) and the quantized histogram (**I**) of ER tracker-red in RKO cells stably transfected with sg-Scb or sg-TP53 and treated with or without oridonin (*n* = 3). Scale bars: 10 μm. **J** Flow cytometry analysis of intracellular ROS levels in RKO cells stably transfected with sg-Scb or sg-TP53 and treated with or without oridonin (*n* = 3). **K** The calcium indicator Fluo-3 AM detects intracellular Ca^2+^ levels in RKO cells stably transfected with sg-Scb or sg-TP53 and treated with or without oridonin (*n* = 6). The statistical results were presented as mean ± SD. Student’s t-test compared the difference in **C**-**D** and **I**-**K**, one-way ANOVA compared the difference in **A**, and two-way ANOVA compared the difference in **E**. * *P* < 0.05, ** *P* < 0.01 compared with ORI (0 μM) or sh-Scb + ORI, and # *P* < 0.05, ## *P* < 0.01 compared with sh-TP53 + DMSO. TP53: tumor protein p53

TCF4 is a key mediator of oridonin-induced ER stress, and its molecular regulation mechanism requires investigation. Multiple tumor studies have reported that TP53 can inhibit the regulation of Wnt/β-catenin dimer on TCF4, preventing the latter from binding to chromosomes and thereby inhibiting the transcription of related target genes [29, 30]. When colorectal cancer cells were treated with varying concentrations of oridonin, the TP53 protein level increased and correlated positively with the oridonin concentration (Fig. 6A). Notably, as the drug concentration increased, the protein concentration of Wnt and β-catenin in colorectal cancer cells gradually decreased (Fig. 6A). In subsequent experiments, TP53 knockdown stable cell lines were utilized to further investigate. In stably TP53 knockdown RKO cells, the expression of Wnt/β-catenin protein and TCF4 protein was increased (Fig. 6B). But after oridonin treatment, the protein level of Wnt/β-catenin decreased again, and the protein level of TCF4 also decreased (Fig. 6B). After treatment with LF3, a specific blocker of β-catenin binding to TCF4, the Wnt/β-catenin protein level did not change significantly, but the expression level of TCF4 was reduced in TP53 knockdown tumor cells, which may result from reduced β-catenin/TCF4 dimer. And the simultaneous treatment with oridonin and LF3 greatly reduced the expression of TCF4 (Fig. 6B). These results demonstrated that oridonin could activate TP53 and regulate the expression of TCF4 in colorectal cancer cells.

To further verify the significance of the TP53/TCF4 axis for the tumor suppressive efficacy of oridonin, we carried out subsequent in vivo and in vitro modeling. Oridonin has no discernible tumor suppressive effect on TP53-knockdown colorectal cancer cells, which is evidenced by the increased proliferation, invasion, and metastasis of tumor cells compared to the control group (Fig. 6C-E). Stably expressing sg-Scb or sg-TP53 colorectal cancer cells were injected into nude mice for subcutaneous modeling, and after two weeks, oridonin was administered intragastrically to nude mice for 14 days (Fig. 6F). Compared with the control group, the volume of subcutaneous xenografts in nude mice in the sg-TP53 group increased faster, but the growth rate of tumor volume was significantly slowed down after oridonin treatment (Fig. S3A). Moreover, subcutaneous tumor grafts of nude mice in the sg-TP53 group had greater endpoint weight and higher fluorescence signal intensity, Ki-67 proliferation index, and CD31-positive microvessels score (Fig. 6G and Fig. S3B-E). Notably, we discovered that after treatment with oridonin, the expression level of TP53 in xenograft tumors increased in both the sg-Scb and sg-TP53 groups (Fig. S3F-G). However, the expression levels of TCF4, FYN, and YOD1 in the subcutaneous xenografts of the sg-TP53 group were all decreased after oridonin treatment, and the expression of CFTR increased (Fig. S3F-G).

In addition, the level of ER stress was also evaluated in in vitro cell models. After inhibiting TP53, oridonin could not cause endoplasmic reticulum swelling and vesicle formation, generate intracellular ROS, and increase calcium ion concentration (Fig. 6H-I). Endoplasmic reticulum morphology, ROS level, and calcium ion concentration were not substantially different between the treatment and control groups.

## Discussion

Oridonin, a natural extract with anti-tumor potential, has been used in the preclinical studies of various cancers [45, 46]. Co-encapsulated nanoparticles of oridonin and cisplatin were also effective in the treatment of NSCLC (Non-Small Cell Lung Cancer) [47]. Our results suggested that oridonin could effectively inhibit the proliferation, migration, and invasion of colorectal tumors. There are many studies on the anticancer mechanism of oridonin. Oridonin can induce apoptosis of oral cancer cells by phosphorylation of histone H2AX in response to DNA damage [48] and inhibition of PI3K/Akt signaling [49]. It can also promote the apoptosis of bladder cancer cells by blocking the expression of TRPM7 through ERK and AKT signaling pathways [50]. In this study, we discovered that oridonin can strongly induce Endoplasmic Reticulum stress, in addition to inducing apoptosis and autophagy in colorectal cancer cells.

The Endoplasmic Reticulum plays a crucial role in protein processing, modification, and folding [20, 51]. However, oncogenic stress can disrupt the protein folding ability of the Endoplasmic Reticulum and trigger ER Stress characterized by the accumulation of misfolded or unfolded proteins [52, 53]. When cells undergo Endoplasmic Reticulum stress, Ca^2+^ is released from the Endoplasmic Reticulum, and ROS production is increased, further causing Endoplasmic Reticulum protein folding dysfunction, disrupting intracellular environmental homeostasis, and inducing apoptosis [42–44]. In this study, we discovered that oridonin increased the ER stress biomarkers ATF4 and CHOP in colorectal tumor cells, as well as intracellular ROS levels, and Ca^2+^ concentration, ultimately inducing apoptosis. Moreover, this activity of oridonin is not unique, since other medicines have also been observed to prevent tumor formation via ER stress. For example, tunicamycin inhibits the N-linked glycosylation of glycoproteins to strongly induce ER stress to induce multidrug-resistant gastric cancer cell death [54]. Microtubule acetylation triggers ER stress in the breast cancer cell to inhibit migration and invasion [55].

Transcription factor 4 (TCF4), a transcriptional activator directly regulated by the Wnt/β-catenin pathway [56], is largely dependent on the Wnt/β-catenin/TCF4 complex for regulating its target genes. Tribbles pseudo-kinase 3 (TRIB3) can then interact with β-catenin/TCF4 to increase stem cell features of colorectal cancer stem cells and tumorigenesis [57]. In our study, TCF4 was identified as a critical regulator of ER stress, and results from both in vivo and in vitro experiments suggested that TCF4 can regulate key ER stress genes, CFTR, FYN, and YOD1. Among these, CFTR induces ER stress by activating the UPR [58]. YOD1 acts as a deubiquitinating enzyme, removing substrate ubiquitination and thereby inhibiting the reverse translocation of unfolded proteins to the Endoplasmic Reticulum, reducing ER stress levels [54]. Moreover, overexpression or silencing of TCF4 caused a decrease or increase in the level of ROS and Ca^2+^ in tumor cells and caused aggregation, tightening, or expansion and swelling of the Endoplasmic Reticulum morphology. And silencing TCF4 reduced proliferation, invasion, and metastasis of colorectal tumor cells, and slowed the growth of xenograft tumors in nude mice, reducing tumor weight, Ki-67 growth index, and tumor CD31-positive neovascularisation. Furthermore, we found that the activation of ER stress by oridonin was attenuated by overexpression of TCF4, which further reduced the inhibitory effect of oridonin on the colorectal tumor. Whereas silencing TCF4 increased the carcinogenic impact of oridonin, it also enhanced oridonin-induced ER stress. The aforementioned evidence revealed that oridonin suppresses colorectal cancer by interfering with the inhibitory impact of TCF4 on ER stress, which further explained the mechanism of oridonin.

TP53 protein is involved in the regulation of cellular processes such as cell cycle arrest, apoptosis, senescence, DNA repair, or metabolic changes, but mutations are detected in most tumors [27, 59]. Oridonin has been reported to upregulate TP53 in a dose- and time-dependent manner and to induce cycle block and upregulation of the apoptosis-related proteins p21 and Bax [60, 61]. Notably, TP53 can prevent β-catenin from binding to TCF4 as a dimer, inhibit its induction of target genes transcriptional activation, and increase cytoplasmic ROS production [29, 62]. In the present study, we also demonstrated the concentration effect of oridonin on TP53. Moreover, Wnt and β-catenin also showed concentration dependence on oridonin. Additionally, we discovered that TP53 was an important part of the oridonin tumor suppressor effect. TP53 knockout could substantially diminish the curative effect of oridonin, increase the expression of TCF4 in in vivo and in vitro, decrease the intensity of ER stress response and ROS level, and preserve calcium homeostasis in colorectal cancer cells.

In summary, we suggested that the TP53/TCF4 axis could play an important role in the process of oridonin-induced Endoplasmic Reticulum stress and that oridonin could induce continuous Endoplasmic Reticulum stress through the regulation of the TP53/TCF4 axis by increasing ROS generation and disrupting Ca^2+^ homeostasis, thus inhibiting tumor progression.

### Mechanism picture


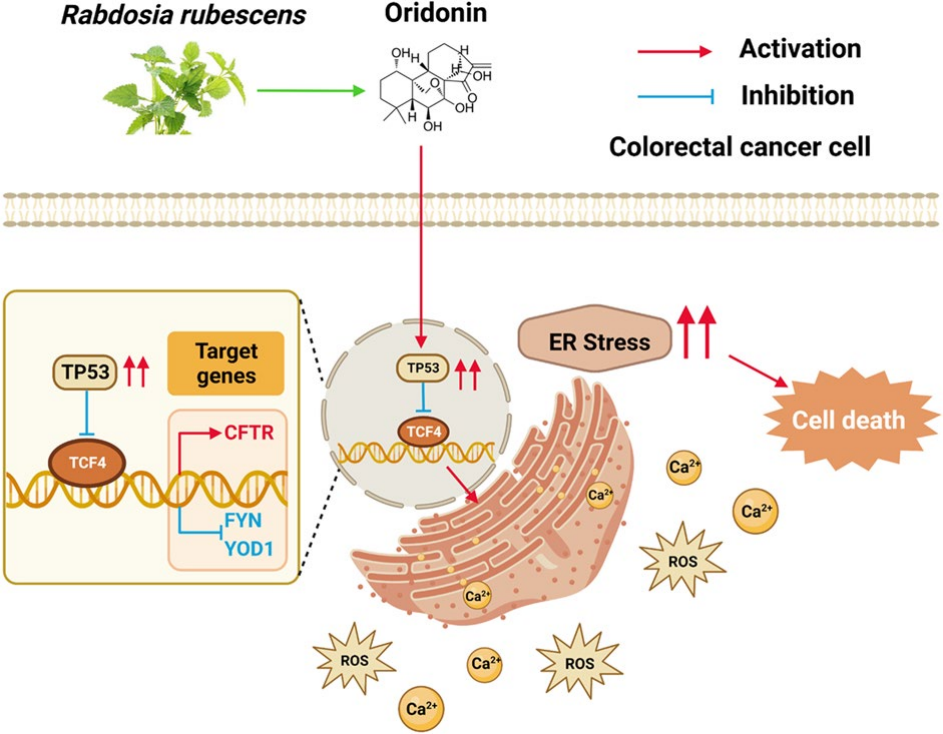


## Conclusion

In conclusion, our study identified TCF4 as an important regulatory factor in the Endoplasmic Reticulum stress and demonstrated that oridonin could activate TP53 to inhibit TCF4 transactivation, thereby continuously enhancing the Endoplasmic Reticulum stress, which ultimately resulted in colorectal cancer cell death. Our study clarified the anti-cancer mechanism of oridonin in greater depth and provided a more solid scientific research basis for promoting the wider clinical application of oridonin. However, there were still some shortcomings in our study. Future pharmacological research and clinical trials are required to investigate the potential of oridonin in clinical application in the future and evaluate its efficacy more comprehensively.

## Supplementary Information

**Additional file 1:** **Table S1. **TCF4 target genes survival and correlation analysis in colorectal cancer.

**Additional file 2:** **Figure S1. **(A-C) Fluorescence images (A), tumor images (B), and weight histogram at the endpoints of xenografts (C) in nude mice bysubcutaneous injection of RKO or LoVo cellsstably expressing Mock, TCF4, sh-Scb, or sh-TCF4 #1 (*n* = 5). The red boxes are representative images of fluorescence andtumor images in Fig. 4E. (D) Immunohistochemical staining (left panels) and the quantitative histogram (right panel) of MKI67 and PECAM1 in the subcutaneous xenografts injection of RKO cells stably transfected with Mock, TCF4, sh-Scb, or sh-TCF4 #1 into the dorsal flanks of nude mice. Scale bars: 100 μm. (E-F) Western blot and qRT-PCR assay showing the levels of TCF4, CFTR, FYN, and YOD1 in the subcutaneous xenografts injection of RKO cells stably transfected with Mock, TCF4, sh-Scb, or sh-TCF4 #1 into the dorsal flanks of nude mice (*n* = 3). Normalized to β-actin. (G-H) Representative fluorescence images for ATF4 (G) and CHOP (H) in RKO or LoVo cells stably expressing Mock, TCF4, sh-Scb,or sh-TCF4 #1 (*n* = 3). Scale bars of G: 50 μm. Scale bars of H: 75 μm. The statistical results were presented as mean ± SD. Student’s t-test compared the difference in C-F. * *P* < 0.05, ** *P* < 0.01 compared with Mock; # *P*< 0.05, ## *P* < 0.01 compared with sh-Scb.MKI67: marker of proliferation Ki-67; PECAM1: platelet and endothelial cell adhesion molecule 1; TCF4: transcription factor 4; CFTR: cystic fibrosis transmembrane conductance regulator; FYN: tyrosine-protein kinase Fyn; YOD1: YOD1 deubiquitinase; ATF4: activating transcription factor 4; CHOP: DNA damage-inducible transcript 3; qRT-PCR: reverse transcription-quantitative polymerase chain reaction.

**Additional file 3:** **FigureS2. **(A-B) Western blot and qRT-PCR assays showing the expression of TCF4, CFTR, FYN, and YOD1 in RKO cells stably transfected with Mock or TCF4 and treated with or without oridonin (*n* = 3). Normalized to β-actin. (C-D) Tumor growth curve (C) and weight at the endpoints (D) of xenografts subcutaneous injection of RKO cells stably transfected with Mock or TCF4 and treatment with or without oridonin (*n* = 5). (E-F) Fluorescence (E) and tumor images (F) of xenografts injection of RKO cells stably transfected with Mock or TCF4 and treatment with or without oridonin. The red boxes are representative images of fluorescence and tumor images in Fig.5D. (*n* = 5). (G) Immunohistochemical staining (left panels) and the quantitative histogram (right panel) of MKI67 and PECAM1 in the subcutaneous xenografts injection of RKO cells stably transfected with Mock or TCF4 and treatment with or without oridonin. Scale bars: 100 μm. (H-I) Western blot and qRT-PCR assay showing the protein levels of TCF4, CFTR, FYN, and YOD1 in subcutaneous xenografts injection of RKO cells stably transfected with Mock or TCF4 and treatment with or without oridonin (*n* = 3). Normalized to β-actin. The statistical results were presented as mean ± SD. Student’s t-test compared the difference in A-B, D, and J-I; two-way ANOVA compared the difference in C. * *P* < 0.05, ** *P* < 0.01 compared with Mock + ORI; # *P* < 0.05, ## *P* < 0.01 compared with TCF4 + DMSO.ORI: oridonin; DMSO: dimethyl sulfoxide.

**Additional file 4:** **FigureS3. **(A-B) Tumor growth curve (A) and weight at the endpoints (B) of xenografts in nude mice by subcutaneous injection of RKO cells stably transfected with sg-Scb or sg-TP53 and treatment with or without oridonin (*n* = 5). (C-D) Fluorescence (C) and tumor images (D) of xenografts by subcutaneous injection of RKO cells stably transfected with sg-Scb or sg-TP53 and treatment with or without oridonin (*n* = 5). The red boxes are representative images of fluorescence and tumor images in Fig. 6G. (E) Immunohistochemical staining (left panels) and the quantitative histogram (right panel) of MKI67and PECAM1 in the subcutaneous xenografts by subcutaneous injection of RKO cells stably transfected with sg-Scb or sg-TP53 and treatment with or without oridonin. Scale bars: 100 μm. (H-I) Western blot and qRT-PCR assay showing the protein levels of TCF4, CFTR, FYN, and YOD1 in subcutaneous xenografts by subcutaneous injection of RKO cells stably transfected with sg-Scb or sg-TP53 and treatment with or without oridonin (*n* = 3). Normalized to β-actin. The statistical results were presented as mean ± SD. Student’s t-test compared the difference in B, E, and G; two-way ANOVA compared the difference in A. * *P* < 0.05, ** *P* < 0.01 compared with sh-Scb + ORI; # *P* < 0.05, ## *P* < 0.01 compared with sh-TP53 + DMSO.TP53: tumor protein p53.

**Additional file 5:** **Figure S4.** (A) Representative images of EdU staining assay in LoVo and RKO cells after treatment with or without oridonin (22 μM) for 24 hours. Scale bars of A: 200 μm. Scale bars of B: 50 μm. (B-C) Representative images of EdU staining and Wound healing assays in colorectal cancer cells stably transfected with Mock, TCF4, or sh-Scb, sh-TCF4 #1. Scale bars: 200 μm. (D-E) Representative images of EdU staining and Wound healing assays in colorectal cancer cells stably transfected with Mock or TCF4 and treated with or without Oridonin. Scale bars: 200 μm. (F-G) Representative images of EdU staining and Wound healing assays in stably expressing sh-Scb or TP53 knockdown colorectal cancer cells treated with or without oridonin. Scale bars: 200 μm.

## Data Availability

The publicly available data are provided in AmiGO 2 and GEO databases.

## Abbreviations

CRC: Colorectal cancer
ORI: Oridonin
ER stress: Endoplasmic reticulum stress
TCF4: Transcription factor 4
TP53: Tumor protein p53
ATF4: Activating transcription factor 4
CHOP: DNA damage-inducible transcript 3, DDIT3
ATF6: Activating transcription factor 6
NFIC: Nuclear factor 1 C-type
STAT3: Signal transducer and activator of transcription 3
CFTR: Cystic fibrosis transmembrane conductance regulator
FYN: Tyrosine-protein kinase Fyn
YOD1: YOD1 deubiquitinase
NF-kappa B: Nuclear factor-kappa B
EIF2AK3/PERK: Eukaryotic translation initiation factor 2 alpha kinase 3
IRE1α: Endoplasmic reticulum to nucleus signaling 1-alpha
MKI67/Ki-67: Marker of proliferation Ki-67
PECAM1/CD31: Platelet and endothelial cell adhesion molecule 1
TRIB3: Tribbles pseudo-kinase 3
ROS: Reactive oxygen species
UPR: Unfolded protein reaction
NSCLC: Non-small cell lung cancer
CCK8 assay: Cell counting kit-8 assay
IC_50_: The half maximal inhibitory concentration
EdU staining: 5-Ethynyl-2'-deoxyuridine staining
qRT-PCR: Reverse transcription-quantitative polymerase chain reaction
HE staining: Hematoxylin–eosin staining
IF: Immunofluorescence
FCM: Flow cytometer
IHC: Immunohistochemistry
DMSO: Dimethyl sulfoxide
Z-VAD: Z-VAD-FMK
CQ: Chloroquine
4-PBA: 4-Phenylbutyric acid
PWMs software: Position Weight Matrices software
GEO database: The Gene Expression Omnibus database
SD: Standard deviation
