# Supplementary material for: Oridonin promotes endoplasmic reticulum stress via TP53-repressed TCF4 transactivation in colorectal cancer
Source: J Exp Clin Cancer Res. 2023 Jun 19;42:150. doi: 10.1186/s13046-023-02702-4 (PMC10278272; doi:10.1186/s13046-023-02702-4)
Supplement: Supplementary file 1 — Additional file 1: Table S1. TCF4 target genes survival and correlation analysis in colorectal cancer. [file 13046_2023_2702_MOESM1_ESM.pdf]

Table S1. TCF4 target genes survival and correlation analysis in colorectal cancer

TCF4	GSE17538			
	Survival		Correlation	
	status (is worse)	p-value	r-value	p-value
AGR2	high	0.107	-0.215	9.75E-04
AQP11	low	0.026	-0.099	0.133
BRSK2	low	0.018	-0.245	1.66E-04
CCND1	high	0.058	-0.104	0.115
CFTR	low	5.00E-04	-0.22	7.35E-04
COPS5	low	0.093	-0.159	0.016
CREB3L3	high	0.102	0.09	0.17
DDX3X	high	0.045	0.067	0.309
DNAJB14	high	0.044	0.329	2.86E-07
EIF2B5	low	0.048	-0.344	7.61E-08
EIF4G1	low	0.065	-0.278	1.68E-05
ELAVL4	low	4.50E-03	0.072	0.278
ERP29	high	7.30E-04	-0.396	4.09E-10
ERP44	high	0.016	-0.178	6.68E-03
FBXO27	low	0.068	-0.081	0.218
FYN	high	2.40E-03	0.539	7.38E-19
GET4	low	0.182	-0.118	0.074
INSIG2	high	1.60E-03	0.26	6.30E-05
MANF	high	0.025	-0.153	0.02
NCK2	low	0.041	-0.026	0.698
NUPR1	low	0.068	0.128	0.052
PARK7	low	0.016	-0.236	2.88E-04
PIK3R1	high	0.1	0.172	8.52E-03
PTPN2	high	2.90E-03	-0.093	0.156
RNF103	low	0.03	-0.022	0.735

RNF183	high	4.30E-04	-0.281	1.45E-05
RNF186	low	7.70E-04	-0.255	8.78E-05
SEL1L	high	0.013	0.19	3.70E-03
SEL1L2	low	4.20E-03	-0.036	0.582
SESN2	low	0.048	-0.08	0.226
SGTA	low	0.065	-0.227	4.82E-04
SGTB	high	8.60E-04	0.132	0.045
TMEM33	low	0.067	-0.353	3.41E-08
TMUB2	low	0.107	0.051	0.438
TRIM25	low	1.40E-03	-0.279	1.57E-05
UBE2J1	low	1.50E-03	0.13	0.047
UGGT1	high	0.073	-0.222	6.72E-04
YOD1	high	1.80E-03	0.524	8.64E-18
**TCF4**	**GSE14333**			
	**Survival**		**Correlation**	
	**status (is worse)**	**p-value**	**r-value**	**p-value**
AGR2	high	0.049	-0.352	7.13E-10
AQP11	low	1.00E-04	-0.086	0.145
BRSK2	low	2.90E-04	-0.221	1.49E-04
CCND1	high	1.90E-03	-0.21	3.23E-04
CFTR	low	5.00E-05	-0.211	2.87E-04
COPS5	high	1.80E-03	-0.270	3.16E-06
CREB3L3	low	3.30E-03	0.143	0.015
DDX3X	low	3.30E-05	0.03	0.608
DNAJB14	high	1.30E-03	0.344	1.83E-09
EIF2B5	low	8.70E-04	-0.471	2.06E-17
EIF4G1	low	0.011	-0.449	8.28E-16
ELAVL4	low	0.042	0.17	3.73E-03
ERP29	low	2.10E-03	-0.401	1.25E-12

ERP44	low	3.00E-03	-0.37	7.25E-11
FBXO27	low	0.04	0.176	2.58E-03
FYN	high	1.60E-03	0.56	2.20E-25
GET4	high	6.80E-03	-0.039	0.513
INSIG2	high	2.70E-05	0.152	9.67E-03
MANF	low	0.024	-0.114	0.053
NCK2	high	0.028	-0.077	0.193
NUPR1	high	0.045	0.223	1.29E-04
PARK7	low	1.10E-03	-0.268	3.83E-06
PIK3R1	low	2.00E-04	0.203	5.01E-04
PTPN2	high	0.031	-0.268	3.63E-06
RNF103	low	0.045	-0.089	0.129
RNF183	low	0.039	-0.202	5.36E-04
RNF186	low	5.40E-04	-0.218	1.80E-04
SEL1L	high	0.025	0.01	0.861
SEL1L2	high	5.70E-03	-0.095	0.106
SESN2	high	0.01	0.041	0.486
SGTA	low	0.015	-0.129	0.028
SGTB	high	7.60E-03	-0.132	0.024
TMEM33	low	0.028	-0.49	5.98E-19
TMUB2	high	8.20E-03	0.101	0.087
TRIM25	low	3.10E-05	-0.174	2.89E-03
UBE2J1	high	0.043	0.053	0.368
UGGT1	low	3.70E-05	-0.213	2.63E-04
YOD1	high	4.50E-03	0.525	6.43E-22

**TCF4**	**GSE33114**			
	**Survival**		**Correlation**	
	**status (is worse)**	**p-value**	**r-value**	**p-value**
AGR2	high	0.066	-0.243	0.011

AQP11	low	3.80E-03	-0.138	0.156
BRSK2	low	0.136	0.032	0.741
CCND1	low	6.10E-03	0.28	3.28E-03
CFTR	low	0.013	-0.162	2.51E-04
COPS5	low	0.464	-0.711	7.07E-18
CREB3L3	high	0.057	-0.052	0.596
DDX3X	high	0.107	-0.440	1.86E-06
DNAJB14	high	0.081	0.474	2.27E-07
EIF2B5	low	0.029	-0.765	5.30E-22
EIF4G1	low	5.80E-04	-0.513	1.33E-08
ELAVL4	high	0.092	0.249	9.44E-03
ERP29	low	0.064	-0.458	6.11E-07
ERP44	low	0.287	-0.271	4.52E-03
FBXO27	high	0.044	0.229	0.017
FYN	high	1.80E-04	0.572	9.66E-11
GET4	low	4.10E-03	0.018	0.855
INSIG2	high	0.022	0.026	0.792
MANF	high	0.107	-0.032	0.743
NCK2	low	0.075	-0.247	9.90E-03
NUPR1	high	1.90E-03	0.701	2.86E-17
PARK7	high	0.109	-0.503	2.95E-08
PIK3R1	low	2.10E-03	0.14	0.147
PTPN2	high	0.074	-0.064	0.513
RNF103	high	0.181	0.506	2.33E-08
RNF183	high	0.206	0.065	0.501
RNF186	low	0.164	0.259	6.70E-03
SEL1L	high	8.50E-03	0.436	2.35E-06
SEL1L2	high	3.90E-03	-0.011	0.912
SESN2	low	0.152	0.247	0.010

SGTA	low	0.016	-0.422	5.50E-06
SGTB	high	0.102	-0.129	0.184
TMEM33	low	0.127	-0.58	4.97E-11
TMUB2	low	0.242	0.185	0.055
TRIM25	low	0.06	-0.199	0.039
UBE2J1	high	0.022	0.595	1.13E-11
UGGT1	low	4.80E-04	-0.33	4.86E-04
YOD1	high	0.015	0.644	5.49E-14
