# Supplementary figures and images for: Oridonin promotes endoplasmic reticulum stress via TP53-repressed TCF4 transactivation in colorectal cancer
Source: J Exp Clin Cancer Res. 2023 Jun 19;42:150. doi: 10.1186/s13046-023-02702-4 (PMC10278272; doi:10.1186/s13046-023-02702-4)

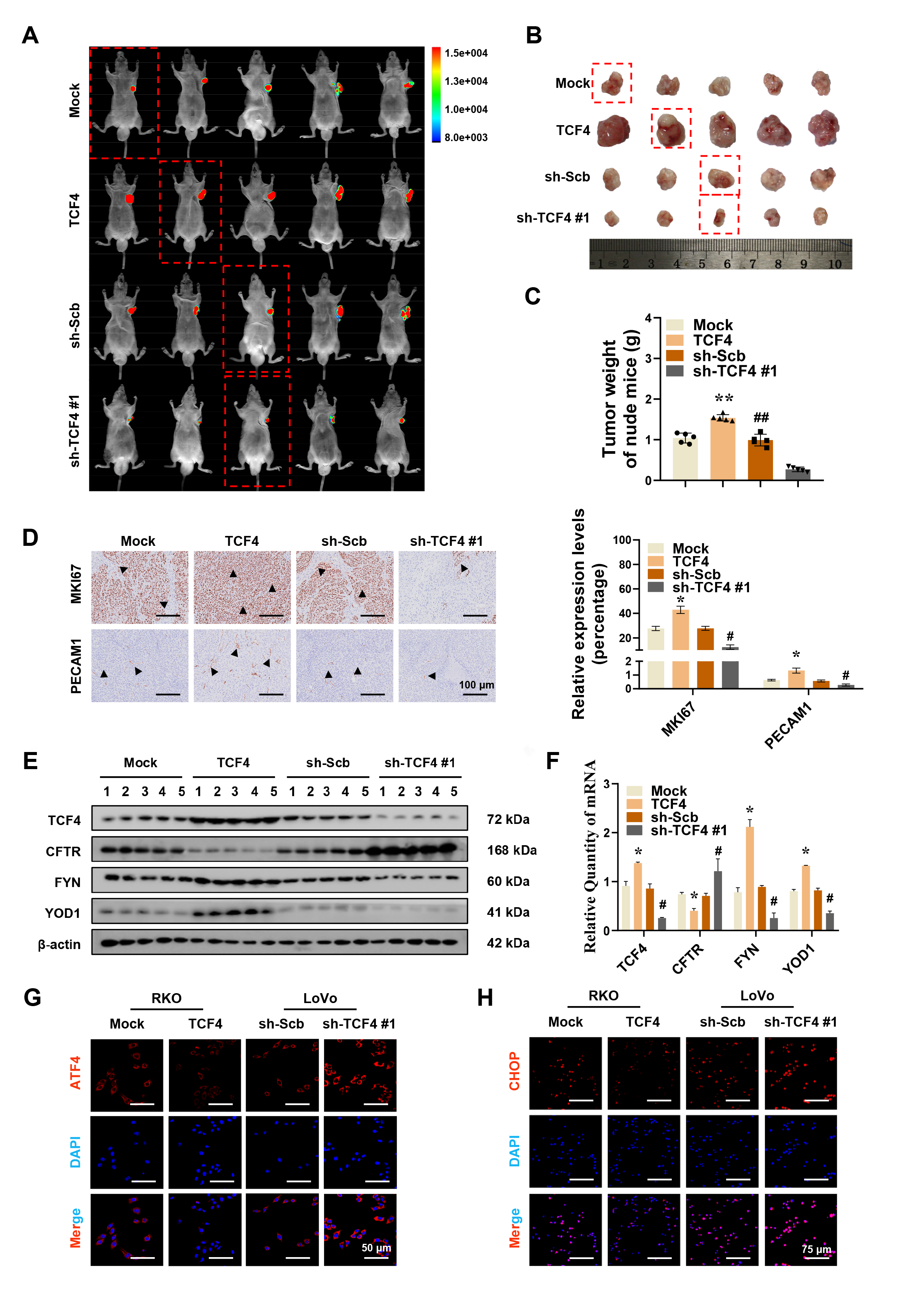

Supplement: Supplementary file 2 — Additional file 2: Figure S1. (A-C) Fluorescence images (A), tumor images (B), and weight histogram at the endpoints of xenografts (C) in nude mice bysubcutaneous injection of RKO or LoVo cellsstably expressing Mock, TCF4, sh-Scb, or sh-TCF4 #1 (n = 5). The red boxes are representative images of fluorescence andtumor images in Fig. 4E. (D) Immunohistochemical staining (left panels) and the quantitative histogram (right panel) of MKI67 and PECAM1 in the subcutaneous xenografts injection of RKO cells stably transfected with Mock, TCF4, sh-Scb, or sh-TCF4 #1 into the dorsal flanks of nude mice. Scale bars: 100 μm. (E-F) Western blot and qRT-PCR assay showing the levels of TCF4, CFTR, FYN, and YOD1 in the subcutaneous xenografts injection of RKO cells stably transfected with Mock, TCF4, sh-Scb, or sh-TCF4 #1 into the dorsal flanks of nude mice (n = 3). Normalized to β-actin. (G-H) Representative fluorescence images for ATF4 (G) and CHOP (H) in RKO or LoVo cells stably expressing Mock, TCF4, sh-Scb,or sh-TCF4 #1 (n = 3). Scale bars of G: 50 μm. Scale bars of H: 75 μm. The statistical results were presented as mean ± SD. Student’s t-test compared the difference in C-F. * P < 0.05, ** P < 0.01 compared with Mock; # P< 0.05, ## P < 0.01 compared with sh-Scb.MKI67: marker of proliferation Ki-67; PECAM1: platelet and endothelial cell adhesion molecule 1; TCF4: transcription factor 4; CFTR: cystic fibrosis transmembrane conductance regulator; FYN: tyrosine-protein kinase Fyn; YOD1: YOD1 deubiquitinase; ATF4: activating transcription factor 4; CHOP: DNA damage-inducible transcript 3; qRT-PCR: reverse transcription-quantitative polymerase chain reaction. [file 13046_2023_2702_MOESM2_ESM.tif]

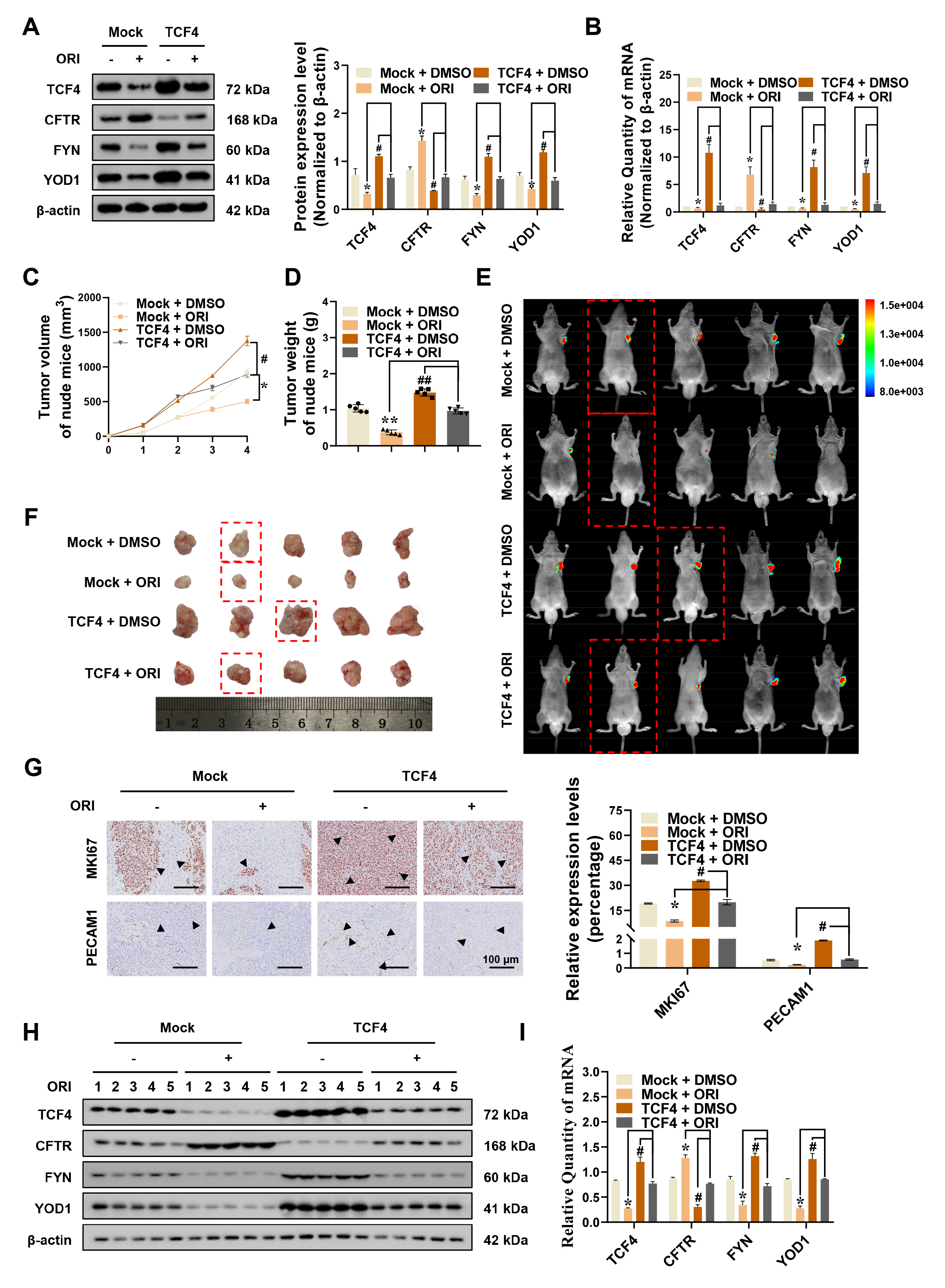

Supplement: Supplementary file 3 — Additional file 3: FigureS2. (A-B) Western blot and qRT-PCR assays showing the expression of TCF4, CFTR, FYN, and YOD1 in RKO cells stably transfected with Mock or TCF4 and treated with or without oridonin (n = 3). Normalized to β-actin. (C-D) Tumor growth curve (C) and weight at the endpoints (D) of xenografts subcutaneous injection of RKO cells stably transfected with Mock or TCF4 and treatment with or without oridonin (n = 5). (E-F) Fluorescence (E) and tumor images (F) of xenografts injection of RKO cells stably transfected with Mock or TCF4 and treatment with or without oridonin. The red boxes are representative images of fluorescence and tumor images in Fig.5D. (n = 5). (G) Immunohistochemical staining (left panels) and the quantitative histogram (right panel) of MKI67 and PECAM1 in the subcutaneous xenografts injection of RKO cells stably transfected with Mock or TCF4 and treatment with or without oridonin. Scale bars: 100 μm. (H-I) Western blot and qRT-PCR assay showing the protein levels of TCF4, CFTR, FYN, and YOD1 in subcutaneous xenografts injection of RKO cells stably transfected with Mock or TCF4 and treatment with or without oridonin (n = 3). Normalized to β-actin. The statistical results were presented as mean ± SD. Student’s t-test compared the difference in A-B, D, and J-I; two-way ANOVA compared the difference in C. * P < 0.05, ** P < 0.01 compared with Mock + ORI; # P < 0.05, ## P < 0.01 compared with TCF4 + DMSO.ORI: oridonin; DMSO: dimethyl sulfoxide. [file 13046_2023_2702_MOESM3_ESM.tif]

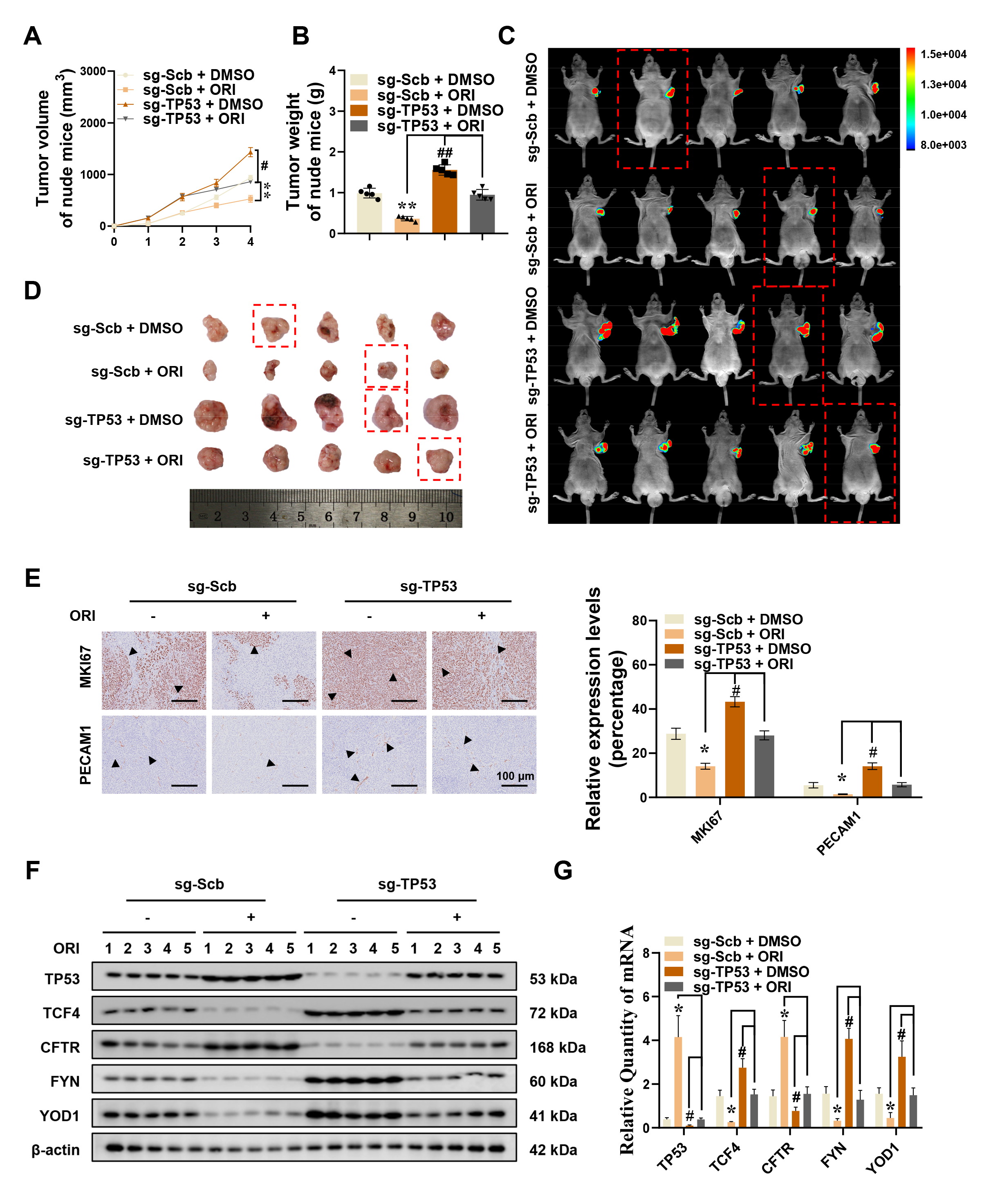

Supplement: Supplementary file 4 — Additional file 4: FigureS3. (A-B) Tumor growth curve (A) and weight at the endpoints (B) of xenografts in nude mice by subcutaneous injection of RKO cells stably transfected with sg-Scb or sg-TP53 and treatment with or without oridonin (n = 5). (C-D) Fluorescence (C) and tumor images (D) of xenografts by subcutaneous injection of RKO cells stably transfected with sg-Scb or sg-TP53 and treatment with or without oridonin (n = 5). The red boxes are representative images of fluorescence and tumor images in Fig. 6G. (E) Immunohistochemical staining (left panels) and the quantitative histogram (right panel) of MKI67and PECAM1 in the subcutaneous xenografts by subcutaneous injection of RKO cells stably transfected with sg-Scb or sg-TP53 and treatment with or without oridonin. Scale bars: 100 μm. (H-I) Western blot and qRT-PCR assay showing the protein levels of TCF4, CFTR, FYN, and YOD1 in subcutaneous xenografts by subcutaneous injection of RKO cells stably transfected with sg-Scb or sg-TP53 and treatment with or without oridonin (n = 3). Normalized to β-actin. The statistical results were presented as mean ± SD. Student’s t-test compared the difference in B, E, and G; two-way ANOVA compared the difference in A. * P < 0.05, ** P < 0.01 compared with sh-Scb + ORI; # P < 0.05, ## P < 0.01 compared with sh-TP53 + DMSO.TP53: tumor protein p53. [file 13046_2023_2702_MOESM4_ESM.tif]

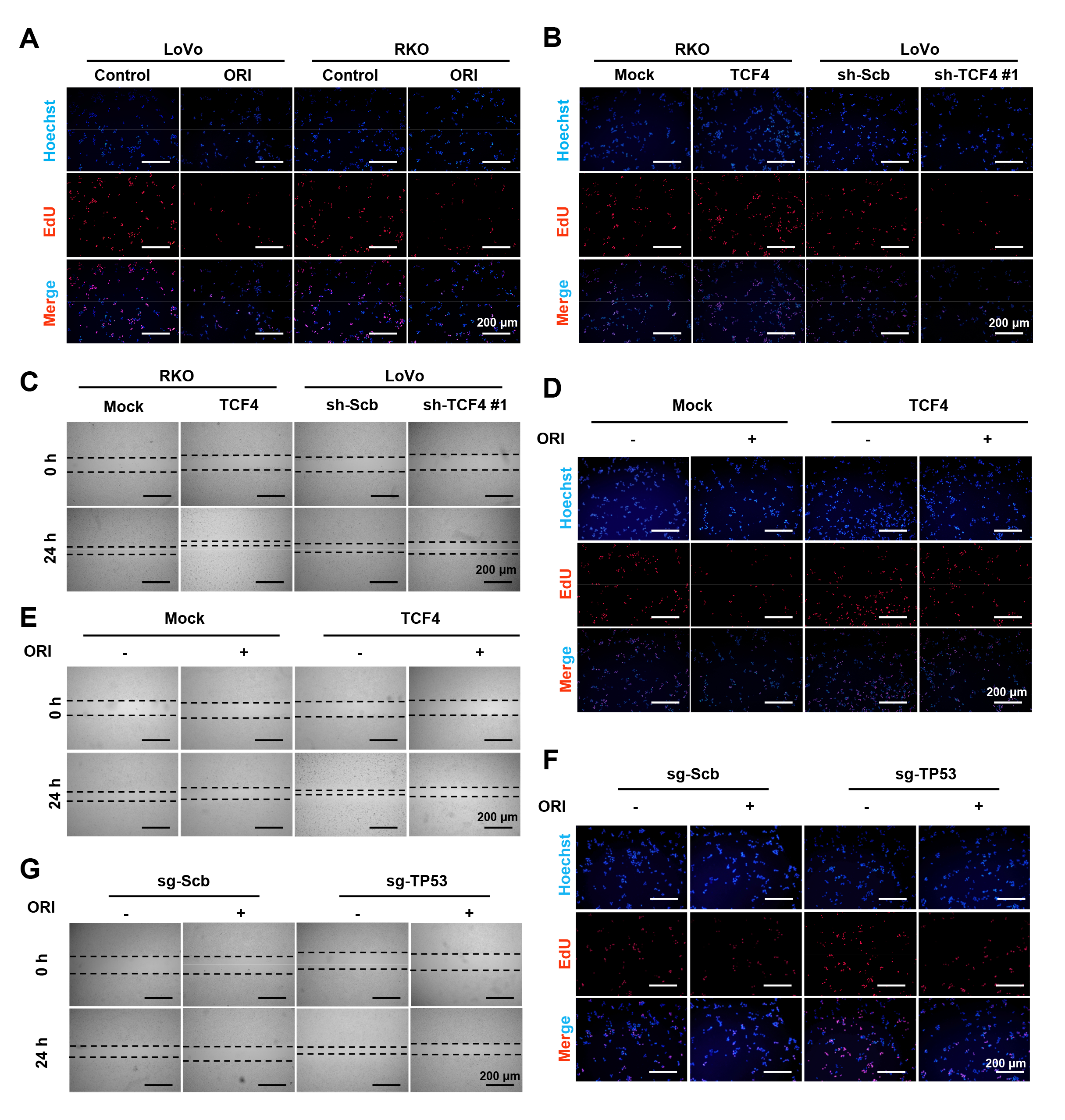

Supplement: Supplementary file 5 — Additional file 5: Figure S4. (A) Representative images of EdU staining assay in LoVo and RKO cells after treatment with or without oridonin (22 μM) for 24 hours. Scale bars of A: 200 μm. Scale bars of B: 50 μm. (B-C) Representative images of EdU staining and Wound healing assays in colorectal cancer cells stably transfected with Mock, TCF4, or sh-Scb, sh-TCF4 #1. Scale bars: 200 μm. (D-E) Representative images of EdU staining and Wound healing assays in colorectal cancer cells stably transfected with Mock or TCF4 and treated with or without Oridonin. Scale bars: 200 μm. (F-G) Representative images of EdU staining and Wound healing assays in stably expressing sh-Scb or TP53 knockdown colorectal cancer cells treated with or without oridonin. Scale bars: 200 μm. [file 13046_2023_2702_MOESM5_ESM.tif]
